# Insights into the Reaction Routes for H_2_ Formation in the Ethanol Steam Reforming on a Catalyst Derived from NiAl_2_O_4_ Spinel

**DOI:** 10.1021/acs.energyfuels.1c01670

**Published:** 2021-07-29

**Authors:** José Valecillos, Sergio Iglesias-Vázquez, Leire Landa, Aingeru Remiro, Javier Bilbao, Ana G. Gayubo

**Affiliations:** Department of Chemical Engineering, University of the Basque Country (UPV/EHU), P.O. Box 644, Bilbao 48080, Spain

## Abstract

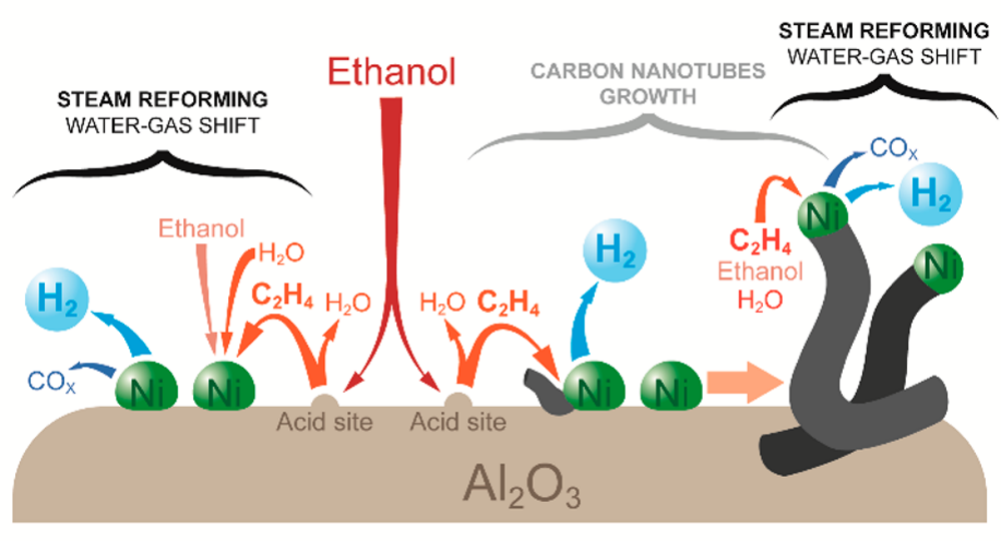

This work describes the satisfactory performance of a Ni/Al_2_O_3_ catalyst derived from NiAl_2_O_4_ spinel in ethanol steam reforming and focuses on studying the prevailing reaction routes for H_2_ formation in this system. NiAl_2_O_4_ spinel was synthesized using a coprecipitation method and reduced at 850 °C to obtain a Ni/Al_2_O_3_ catalyst. The spinel structure and catalyst were characterized using XRD, TPR, N_2_ physisorption, NH_3_ adsorption and TPD, TPO, SEM, and TEM. The experiments were carried out in a fluidized-bed reactor at 500 or 600 °C and different space-time values, using pure ethanol, ethanol–water, pure ethylene, or ethylene–water feeds. The reaction takes place through two paired routes activated by each catalyst function (metal and acid sites) whose extent is limited by the selective catalyst deactivation. The results evidence that at the beginning of the reaction the main route for the formation of H_2_ and carbon (nanotubes) is the dehydration of ethanol on acid sites followed by decomposition of ethylene on the Ni–Al_2_O_3_ interface. This route is favored at 500 °C. After the rapid deactivation of the catalyst for ethylene decomposition, the route of H_2_ formation by steam reforming of ethanol and water gas shift reactions over Ni sites is favored. The morphology of the carbon deposits (nanotubes) allows the catalyst to maintain a notable activity for the latter pathways, with stable formation of H_2_ (during 48 h in the experiments carried out). At 600 °C, the extent of the gasification reaction of carbon species lowers the carbon material formation. The high formation of carbon material is interesting for the coproduction of H_2_ and carbon nanotubes with low CO_2_ emissions.

## 1 Introduction

Transition toward an energy model from renewable and low carbon footprint sources is one of the sustainable development goals. In this context, H_2_ is an alternative energy vector, because it is a clean fuel (its combustion to generate energy only yields water) and can be produced from renewable raw materials.^1^ Among different options, the ethanol steam reforming (ESR) is a promising alternative for the sustainable H_2_ production, provided that ethanol can be obtained from biomass (bioethanol).^2^ Additional advantages of using bioethanol as a feedstock in steam reforming processes are its high hydrogen content, ease of handling, and low toxicity as well as no need to separate water. The presence of water limits the catalyst activity for other bioethanol valorization routes such as the production of ethylene and hydrocarbons.^3^

The ESR reaction, eq 1, yields H_2_ and CO, and the water–gas shift reaction, eq 2, also takes place at steam reforming conditions yielding H_2_ and CO_2_, giving the global stoichiometry of eq 3. However, the high reactivity of ethanol gives way to a complex reaction mechanism involving other parallel reactions,^4,5^ such as the dehydrogenation, eq 4, dehydration, eq 5, and decomposition, eq 6. Subsequently, the products of these reactions are also reactive giving way to acetaldehyde steam reforming, eq 7, decomposition, eq 8, ethylene steam reforming, eq 9, conversion into H_2_ and carbon material, eq 10, CO disproportionation (Boudouard reaction), eq 11, methanation, eq 12, and methane steam reforming (reverse of this equation) reactions.
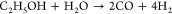
1
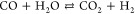
2
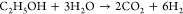
3
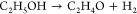
4
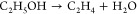
5
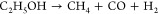
6
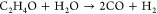
7
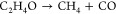
8
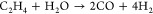
9
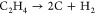
10
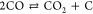
11
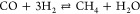
12

The effect of the conditions (particularly the temperature and steam/ethanol ratio) on the extent of these reactions is determined by the thermodynamics, and this is extensively reported in the literature.^6−9^ The increase in the temperature favors steam reforming reactions, eqs 1, 3, 7, and 9 because of their endothermic nature. However, it partially disfavors the extent of the water–gas shift, eq 2, CO disproportionation, eq 11, and methanation, eq 12, reactions because of their moderate exothermic nature. Likewise, the increase in the steam/ethanol ratio favors an equilibrium shift toward product formation when water is a reactant, eqs 1–3, 7, and 9. The increase in the temperature and water concentration in the reaction medium also favors carbon gasification, eq 13, and the reverse of eq 11. The increase in the space time, defined as the ratio between the catalyst weight and reactant flow rate, would favor the extent of catalytic reactions.^10,11^
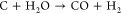
13

The common catalysts for the ESR are based on Co or Ni supported on different materials because of their high activity for breaking C–C bonds and low cost compared to noble metals.^12−16^ Co is more active than Ni at low temperature values, enhancing the water–gas shift reaction, eq 2, and consequently boosting the H_2_ formation. The reaction routes favored on both catalysts comprise the ethanol dehydrogenation, eq 4, and subsequent acetaldehyde steam reforming, eq 7.^14^ However, the Ni capacity to break C–C bonds is higher than that of Co,^14,17,18^ favoring reaction routes based on the ethanol decomposition reaction, eq 6, and subsequent methane steam reforming (reverse of eq 12). Likewise, Tian et al.^19^ demonstrated that the dominant presence of reduced Ni crystals favors the H_2_ formation, whereas Ni^2+^ promotes CH_4_ formation. Furthermore, the metal size affects the catalyst performance, and generally, small metal sizes enhance the activity and selectivity for steam reforming reactions and lowers coke deposition.^18^ Another peculiar characteristic of Ni is the formation of carbon nanotubes in the ESR.^20,21^ The formation of these materials is interesting for boosting the process feasibility, because carbon nanotubes are relevant materials for many applications in different fields, from photonics to catalysis.^22,23^

The support properties similarly affect the catalytic performance in the ESR.^24^ The common supports are based on acid or basic oxides or a combination of both, without or with the addition of promoters. Al_2_O_3_ is often preferred because of its high mechanical properties and surface area and acid properties that enhance the metal–support interactions and ethanol conversion. However, the Al_2_O_3_ acid sites also catalyze the ethanol dehydration, eq 5, yielding ethylene that undergoes decomposition to yield carbon material and H_2_ through eq 10.^25^ The use of basic supports or the combination of basic oxides with Al_2_O_3_, such as La_2_O_3_, CeO_2_, ZnO, or ZrO_2_, improves the dispersion, prevents the sintering of the metal particles, and favors some reactions boosting the H_2_ yield.^25−34^ The presence of these oxides improves the oxygen mobility and the H_2_O adsorption and dissociation, resulting in promoting steam reforming reactions, eqs 1, 3, 7, and 9, the water–gas shift reaction, eq 2, and coke gasification, eq 13. The addition of basic promoters, such as CaO, reduces the support acidity and decreases the interaction between the metal precursor and the support, thus enhancing the formation of the metal particles onto the support.^35^ The neutralization of the support acid sites avoids the ethanol dehydration forming ethylene, eq 5, that is a relevant coke precursor. The use of CaO also captures CO_2_ by carbonation from the reaction medium, leading to a shift of the equilibrium of the water–gas shift reaction, eq 2, thus increasing the H_2_ yield.^36,37^

The catalyst stability is a key feature for the feasibility of ESR processes at an industrial scale, and catalysts based on spinel structures are a promising option for this objective. Barroso et al.^38^ observed the moderate activity of NiAl_2_O_4_, CuAl_2_O_4_, and ZnAl_2_O_4_ spinel structures directly used for the ESR at 500 °C. Likewise, Muroyama et al.^13^ compared the activity of Ni-based spinel structures with different secondary sites (NiAl_2_O_4_, NiFe_2_O_4_, or NiMn_2_O_4_) in the ESR at 550 °C, finding that the NiAl_2_O_4_ spinel structure is more stable. Subsequent works have proved that the reduction of NiAl_2_O_4_ spinel at 850 °C generates a Ni/Al_2_O_3_ catalyst with high activity for bio-oil steam reforming (BSR).^39,40^ This catalyst has a high dispersion of reduced Ni crystals and can be regenerated upon coke combustion, recovering its initial activity. Nuñez Meireles et al.^41^ used Ni_1–*x*_Cu_*x*_Al_2_O_4_ (*x* = 0, 0.01, 0.05, 0.1) spinel structures to generate in situ reduced Ni crystals at low reduction temperature values (500 °C), finding that the Cu addition benefits the exsolution of Ni to the surface of the spinel structure, improving the dispersion of the resulting catalyst. The catalysts yielded up to 70% of H_2_ in the ESR, with no acetaldehyde, ethylene, or ethane observed, and therefore obtaining low carbon formation.

The objective of this work is to study the mechanism of the ESR over a Ni/Al_2_O_3_ catalyst derived from NiAl_2_O_4_ spinel. This knowledge would guide the selection of suitable conditions for obtaining a high purity H_2_ stream with high stability. The choice of this catalyst is based on our previous experience in the bio-oil steam reforming, in which it showed an outstanding performance and good regeneration capacities.^39,40,42,43^ With the purpose of analyzing the extent of different reactions, the experiments were carried out at different space-time values at 500 and 600 °C. Additionally, the experiments were carried out in a fluidized-bed reactor that guarantees the catalytic bed isothermal operation and avoids flow blockage problems despite the presence of a high solid carbon content. Consequently, the use of this reactor facilitates the interpretation of the results for studying the reaction mechanism. The comparison of the results of evolution with time on stream of the distribution of products in the conversion of ethanol and ethylene, with and without water, and the study of the content, nature (carbon nanotubes), and rate of deposition of carbonaceous material allow the following to be distinguished: (i) the evolution with time of the relative importance of the H_2_ formation routes, via dehydration or ethanol reforming, (ii) the rapid selective deactivation of the catalyst for the first route, and (iii) the subsequent stability of the catalyst. This stability is a characteristic of the catalyst derived from NiAl_2_O_4_ spinel.

## 2 Experimental Section

### 2.1 Catalyst Synthesis

The NiAl_2_O_4_ spinel precursor was synthesized using the coprecipitation method.^40,44−47^ First, an aqueous solution of metallic precursors was prepared with Ni(NO_3_)_2_·6H_2_O (Panreac, 99%) and Al(NO_3_)_3_·9H_2_O (Panreac, 98%) and a Ni loading of 33 wt % (stoichiometric value for the coprecipitation reaction). Then, a 0.6 M NH_4_OH (Fluka, 5 M) solution was added dropwise to the metallic precursor solution until reaching a pH of 8 with constant stirring. The precipitate was recovered through filtration and washing with distilled water to eliminate the excessive ammonium ions. NiAl_2_O_4_ spinel was obtained upon drying the recovered precipitate at 110 °C for 24 h and calcination at 850 °C for 4 h with a ramp of 10 °C min^–1^. Finally, we obtained the Ni/Al_2_O_3_ catalyst upon reducing the NiAl_2_O_4_ spinel precursor at 850 °C for 4 h in a H_2_–N_2_ flow (10 mol % H_2_) in the reaction system described in Section 2.3.

For comparison, a supported Ni/Al_2_O_3_ catalyst was also prepared by wet impregnation as described elsewhere.^48^ A commercial γ-Al_2_O_3_ was contacted with a Ni(NO_3_)_2_·6H_2_O (Panreac, 99%) solution in a rotatory evaporator, followed by drying at 110 °C for 24 h and calcination at 600 °C for 4 h in order to obtain the NiO/Al_2_O_3_ precursor. The Ni/Al_2_O_3_ was obtained upon reducing the NiO/Al_2_O_3_ under the same conditions as the NiAl_2_O_4_ spinel (850 °C for 4 h in a H_2_–N_2_ flow (10 mol % H_2_)). The nominal Ni loading was 33 wt % (the same as in the NiAl_2_O_4_ spinel).

### 2.2 Catalyst Characterization

The textural properties of the precursors and catalysts were determined using N_2_ physisorption at −196 °C in a Micromeritics ASAP 2010 analyzer. A typical measurement consisted of outgassing a sample (∼200 mg) at 150 °C and under vacuum (10^–3^ mm Hg) followed by a period of N_2_ adsorption at 77 K with increasing relative pressure values (from 0 to 1) and N_2_ desorption at decreasing relative pressure values (from 1 to 0). We used the equilibria data to calculate the specific surface area using the BET method and the average pore size using the BJH method.

The reduction features of NiAl_2_O_4_ spinel and NiO/Al_2_O_3_ precursors were determined by carrying out a temperature-programmed reduction (TPR) measurement in a Micromeritics AutoChem 2920 analyzer. The measurement consisted of outgassing a sample (∼200 mg) in a U-shaped reactor at 200 °C in He flow, then cooling down to room temperature, switching the flow to a H_2_–Ar mixture (10 mol % H_2_), and heating up to 950 °C at 10 °C min^–1^. The H_2_ uptake was measured with a thermal conductivity detector (TCD).

The structural properties were determined using X-ray diffraction (XRD). The measurements were carried out in a Bruker D8 Advance diffractometer with a Cu Kα1 radiation. The equipment is provided with a germanium primary monochromator, Bragg–Brentano geometry, and with a Cu Kα1 wavelength of 1.5406 Å, corresponding to an X-ray tube with a Cu anticathode. A Sol-X dispersive energy detector was used, with a window optimized for Cu Kα1 for limiting the fluorescence radiation. Data collection was taken continuously, from 10 to 80° with a step of 0.04° in 2θ and measurement time of 10 min. We used the Scherrer equation to determine the crystallite size of reduced Ni crystals.

The acid properties of the catalysts were determined using NH_3_ adsorption and temperature-programmed desorption (NH_3_-TPD). First, the amount of adsorbed NH_3_ was measured in a TA Instruments SDT 2960 Simultaneous DTA-TGA thermobalance. The measurement consisted of outgassing a sample (∼30 mg) at 800 °C in N_2_ flow, then cooling down to and stabilizing at 150 °C, pumping NH_3_ at 0.75 mL min^–1^ until observing saturation (constant weight gain), and subsequent sweeping with N_2_ until stabilizing the sample weight. The difference between the initial and final sample weight is the amount of chemisorbed NH_3_ attributable to the presence of acid sites in the sample. The NH_3_-TPD profile was obtained in a Micromeritics AutoChem 2920 analyzer coupled with a Pfeiffer Vacuum mass spectrometer (MS). The measurement consisted of outgassing a sample (∼100 mg) at 800 °C in He flow, then cooling down to and stabilizing at 150 °C, pumping NH_3_ at 0.75 mL min^–1^ until stabilization, and heating up to 800 °C at 5 °C min^–1^. The profile of desorbed NH_3_ is followed using the TCD signal and the *m*/*z* signal of 15 in the MS, to avoid masking problems with other *m*/*z* signals corresponding to NH_3_ (16 and 17) when water is present in the desorption effluent.^49^

The amount and nature of the carbon material formed was determined using temperature-programmed oxidation (TPO) measurements in a TA Instruments TGA Q5000TA IR thermobalance. A typical measurement consisted of stabilizing a used catalyst sample (∼20 mg) at 100 °C in an air flow (8.5 mol % O_2_) and heating up to 850 °C at 2 °C min^–1^. The TPO profile is obtained from the derivative of the thermogravimetric signal (DTG), and the coke content is calculated from the area under the TPO profile in the region of coke combustion. The morphology of this carbon material was analyzed using scanning electron microscopy (SEM) and transmission electron microscopy (TEM). The SEM images were obtained in a Hitachi S-4800N field emission gun scanning electron microscope (FEG-SEM) using an accelerating voltage of 5 kV. For the TEM analysis, the samples were dispersed in ethanol at a concentration of 4 mg mL^–1^. A sample of the dispersion (3 μL) is placed on a grid covered with a carbon film and, after allowing to dry, the TEM images were obtained in a JEOL 1400 Plus transmission electron microscope using an accelerating voltage of 100 kV.

### 2.3 Catalytic Tests

The ESR experiments were carried out in a reaction equipment (Microactivity reference-PID Eng & Tech) provided with an isothermal fluidized-bed reactor (22 mm internal diameter and total length of 460 mm), coupled online with an Agilent 3000 microgas chromatograph (micro-GC) through a thermally insulated line, for the analysis of the gaseous effluent from the reactor. The micro-GC has four column modules for the detection and quantification of the reaction components: (1) molecular sieve capillary column for separating O_2_, N_2_, H_2_, CO, and CH_4_; (2) PLOT Q capillary column for separating light oxygenates and hydrocarbons (C_1_–C_3_), CO_2_, and water; (3) alumina capillary column for separating C_2_–C_4_ hydrocarbons; (4) Stabilwax type column for separating oxygenates (C_2+_) and water. The atom balance (C, H, O) is closed in all the experiments above 95%.

The catalytic bed consisted of an inert material (SiC from VWR Chemicals sieved at 105 μm) and a preset catalyst amount (depending on the desired space time), keeping an initial height/diameter ratio above 2 for all the experiments. The use of a fluidized bed allows an isothermal operation to be kept even with a high content of carbon material. The feed consisting of an ethanol–water mixture or pure ethanol was pumped and mixed with a N_2_ flow (diluent and carrier) and evaporated prior to entering the reactor. A constant ethanol flow rate of 2 g h^–1^ was pumped in all the experiments, and the N_2_ and water flow rates were adjusted according to the desired steam/ethanol ratio (balance calculations for N_2_) to keep a constant ethanol concentration of 5 mol % in all the experiments. The total flow rate (324.6 mL min^–1^ at 0 °C and 1 atm) gives an upward gas linear velocity of about 3 times the minimum fluidization velocity of the catalytic bed at reaction conditions. We changed the space time (ratio between the catalyst weight and ethanol flow rate in g h g^–1^, hereon simplified as h) by changing the catalyst weight. The reaction temperature used was 500 and 600 °C. Additionally, we carried out experiments with an ethylene feed at a gas flow rate equivalent to that of ethylene formed from ethanol (molar flow rate assuming a complete ethanol conversion into ethylene). In these experiments with ethylene, water was pumped at the calculated flow rate according to the desired steam/ethylene ratio.

The catalytic performance was analyzed calculating the ethanol or ethylene conversion (*X*)
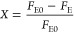
14where *F*_E0_ is the ethanol (or ethylene when this is the feed) molar flow rate at the reactor inlet, and *F*_E_ is the flow rate at the reactor outlet. Likewise, we calculated the product i yield (*Y*_i_) as
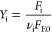
15where *F*_i_ is the product i molar flow rate in the reactor outlet, and ν_i_ is the stoichiometric factor between product i and ethanol (or ethylene), whose values are 6 for H_2_, 2 for CO_2_, CO, and CH_4_, and 1 for acetaldehyde and ethylene. The product i selectivity (*S*_i_) is
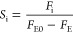
16

Additionally, we did calculations of the thermodynamic equilibrium for the ESR reaction at the conditions used for the experiments. The calculations were carried out in the process simulation software PRO/II 10.1 using a Gibbs reactor system so that the equilibrium calculations are based on the Gibbs energy minimization (nonstoichiometric approach). The components considered in the reaction medium were those in the feed (ethanol, water, and N_2_), H_2_, and carbon products (ethylene, CO_2_, CO, CH_4_, and C (graphite)).

## 3 Results

### 3.1 Spinel and Catalyst Properties

**Figure 1:**
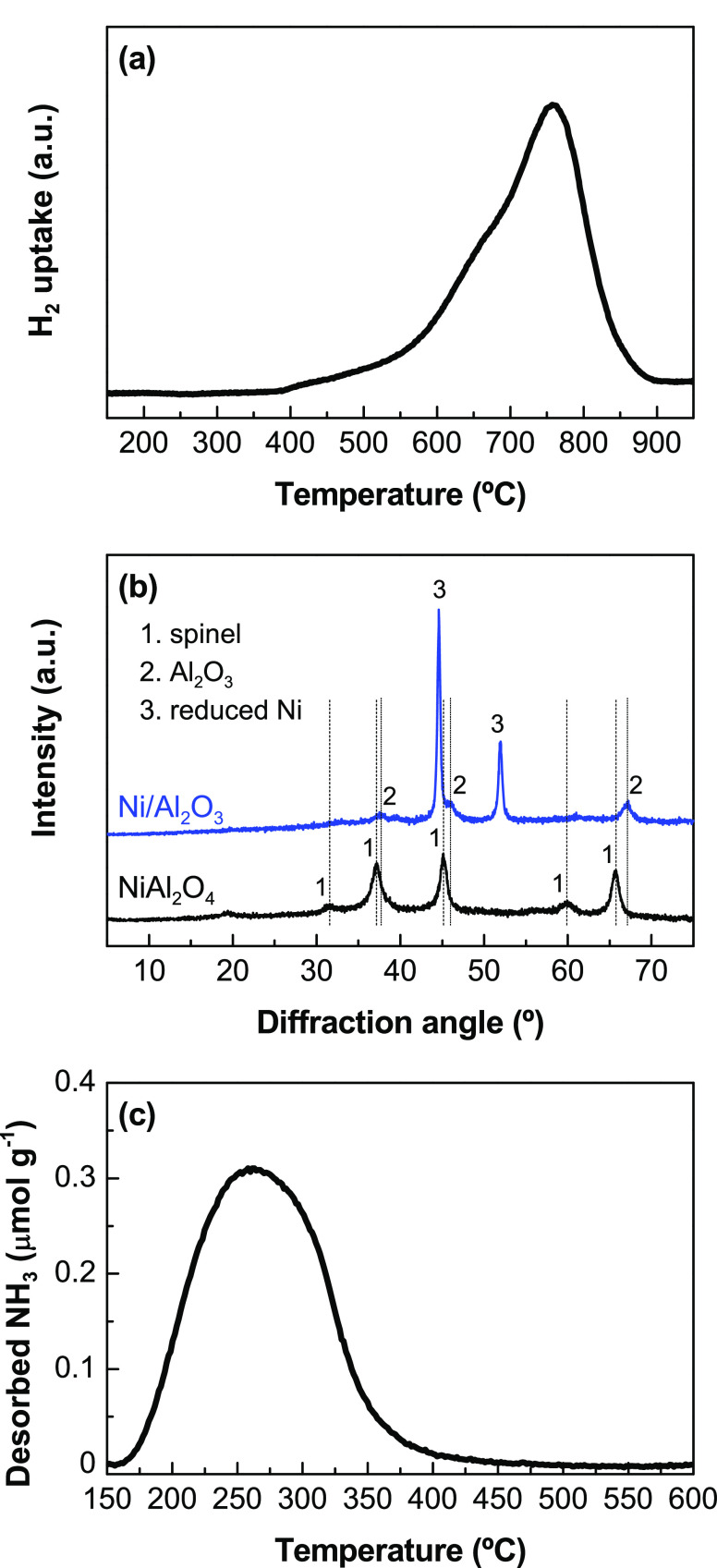
Characterization results: (a) TPR profile of NiAl_2_O_4_ spinel, (b) XRD patterns of NiAl_2_O_4_ spinel and corresponding derived Ni/Al_2_O_3_ catalyst, and (c) NH_3_-TPD profile of the Ni/Al_2_O_3_ catalyst.

The TPR profile of NiAl_2_O_4_ spinel (Figure 1a) indicates that the maximum H_2_ uptake takes place at 760 °C, corresponding to the reduction of Ni species incorporated in the spinel structure.^39,41,50^ In contrast, the TPR profile of the NiO/Al_2_O_3_ precursor prepared by wet impregnation (Figure S1a) exhibits two broad peaks at lower temperature values corresponding to the reduction of NiO_*x*_ species with different interactions with the support, with no evidence for the formation of spinel phases due to the low calcination temperature (600 °C). Both NiAl_2_O_4_ spinel and NiO/Al_2_O_3_ precursors show similar total H_2_ uptake according to their similar Ni loading.

Likewise, the XRD pattern of NiAl_2_O_4_ spinel (Figure 1b) indicates the formation of the typical cubic structure expected for this spinel,^47^ showing intense peaks at 2θ = 37.2, 45.2 and 65.7° (JCPDS 78-1601).^51^ Upon reduction of the NiAl_2_O_4_ spinel structure, the XRD pattern of the Ni/Al_2_O_3_ catalyst (Figure 1b) shows peaks at 2θ = 44.6 and 52.0° corresponding to reduced Ni crystals and at 2θ = 37.5, 46.0, and 67.1° (JCPDS 01-074-2206),^52^ corresponding to Al_2_O_3_ phases. This indicates that the reduction process at 850 °C completely converted NiAl_2_O_4_ spinel into reduced Ni crystals supported on Al_2_O_3_ (Ni/Al_2_O_3_), as previously reported in other works.^40,41,50^ We used the Scherrer equation to estimate the mean reduced Ni crystal size using the diffraction peak at 2θ = 52° and obtained a mean size of 26 nm for the batch of NiAl_2_O_4_ spinel reduced in the reactor. In comparison, the XRD pattern of the NiO/Al_2_O_3_ precursor (Figure S1b) evidences the predominant presence of NiO phases (2θ = 37.5, 43.5, and 63.1°, JCPDS 78-0643), with no evidence for the formation of spinel phases, and upon reduction, these NiO phases are converted into reduced Ni phases (with a mean crystal size of 50 nm) supported on Al_2_O_3_. These results evidence that the use of NiAl_2_O_4_ spinel as catalyst precursor improves the dispersion of Ni crystals in supported catalysts with high Ni loadings.

Table 1 summarizes the textural properties of NiAl_2_O_4_ spinel and corresponding derived Ni/Al_2_O_3_ catalyst determined with N_2_ physisorption. The specific surface area of the spinel (94 m^2^ g^–1^) is similar to the value obtained by Morales-Marin et al.^50^ for a NiAl_2_O_4_ spinel prepared by the same method (98 m^2^ g^–1^). The Ni/Al_2_O_3_ catalyst has lower specific surface area than the NiAl_2_O_4_ spinel structure, whereas it has a higher pore volume and larger mean pore size. In comparison, the NiO/Al_2_O_3_ precursor and corresponding derived Ni/Al_2_O_3_ catalyst show lower specific surface area and higher pore size (Table S1).

**Table 1:** Textural Properties of the NiAl_2_O_4_ Spinel Structure and Derived Ni/Al_2_O_3_ Catalyst

property	NiAl_2_O_4_	Ni/Al_2_O_3_
*S*_BET_ (m^2^ g^–1^)	94.0	68.2
*V*_pore_ (cm^3^ g^–1^)	0.217	0.228
pore size (nm)	7.94	12.5

The Ni/Al_2_O_3_ catalyst obtained from NiAl_2_O_4_ spinel showed capacity to adsorb NH_3_ giving a density of acid sites of 37.7 μmol g^–1^, evidencing the formation of acidic Al_2_O_3_ phases upon the NiAl_2_O_4_ spinel reduction^53^ without ruling out the contribution of Ni sites to the acidity.^54^ Surprisingly, the Ni/Al_2_O_3_ catalyst obtained from the NiO/Al_2_O_3_ precursor prepared through wet impregnation showed a lesser density of acid sites (10.7 μmol g^–1^). The TPD profiles (Figures 1c and S1c) show that NH_3_ desorbs between 150 and 350 °C, corresponding to weak–medium strength acid sites.^55^ This presence of acid sites on the surface of both Ni/Al_2_O_3_ catalysts provides a bifunctional catalyst with acid and metal sites, with higher acid site density and better Ni crystal dispersion in that derived from NiAl_2_O_4_ spinel.

### 3.2 Gaseous Products of ESR

**Figure 2:**
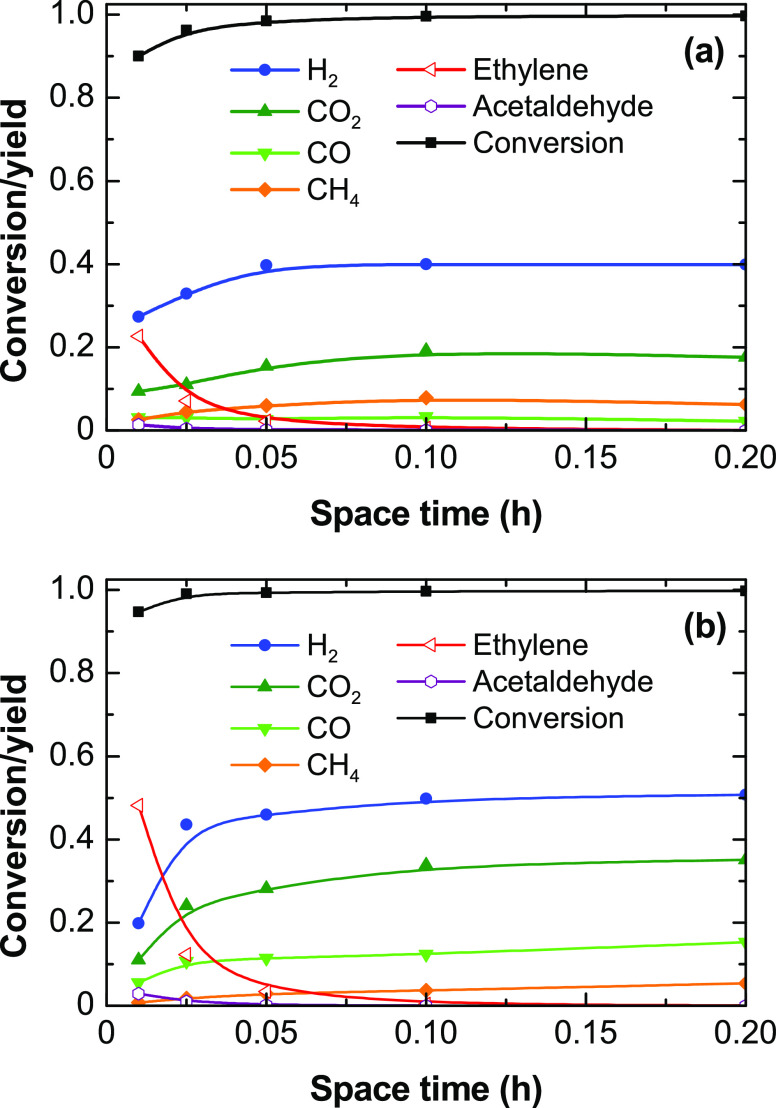
Evolution with space time of the ethanol conversion and product yields for the ESR at (a) 500 and (b) 600 °C. Reaction conditions: ethanol partial pressure, 0.05 bar, steam/ethanol/N_2_ molar ratio, 3:1:16.

Figure 2 shows the evolution with space time of the initial ethanol conversion and product yields at 500 °C (Figure 2a) and 600 °C (Figure 2b). The conversion rapidly increases with the increase in the space time, showing complete conversion at relatively low space-time values (above 0.025 h at 500 °C and above 0.01 h at 600 °C). The main products are H_2_, CO_2_, CO, CH_4_ and ethylene, with traces of ethane and acetaldehyde. At both temperature values, the H_2_, CO_2_, CO, and CH_4_ yields increase with increasing space-time values, indicating these are final products in the reaction scheme, whereas the ethylene yield notably decreases with increasing space-time values, indicating ethylene is an intermediate. Likewise, the acetaldehyde yield decreases with increasing space-time values, indicating the role of acetaldehyde as an intermediate, though its formation, eq 4, is less favored than that of ethylene. Thus, the increase in the space time favors the extents of reactions leading to the formation of H_2_, CO_2_, CO, and CH_4_ from ethanol or intermediates (ethylene and acetaldehyde). The ethylene formation occurs by means of the ethanol dehydration, eq 5, catalyzed on acid sites,^12,56,57^ probably provided by the Al_2_O_3_ phase of the catalyst. The reaction rates, inferred from the variations in the conversion and product yields with space time, are faster at 600 °C than at 500 °C, which is an expected observation for the steam reforming of oxygenates.^58^ The H_2_, CO_2_, CO, and ethylene yields are higher at 600 °C than at 500 °C, whereas the CH_4_ yield is lower, because the methane reforming reaction is thermodynamically favored at high temperatures.^6,7^

To compare with the experimental results, Table 2 summarizes the calculated yields of the thermodynamic equilibrium for the ESR, assuming graphite as carbon representation (the one in the database used for the calculations that is structurally close to the carbon material). The values in Table 2 indicate that the increase in the temperature increases the equilibrium yields of H_2_ and CO, slightly decreases the CO_2_ yield, and significantly decreases the CH_4_ yield. These equilibrium calculations give evidence that the experimental results at 500 °C and high space-time values do not correspond to the thermodynamic equilibrium (Table 2), although the H_2_ yield (0.40) is close to the equilibrium yield at this temperature. At 600 °C, the difference between experimental and equilibrium results is greater. Curiously, the experimental CO_2_ yield does not follow the expected trend from the thermodynamic calculations; in general, the CO_2_ yield is below the equilibrium value and is higher at 600 °C than at 500 °C (opposite to the equilibrium calculations). This indicates that the H_2_ formation routes significantly vary depending on the temperature: H_2_ formation through ethanol or ethylene steam reforming, eqs 1 and 9, or through ethanol decomposition, eq 6, seem to be favored at 600 °C, whereas H_2_ formation through ethylene decomposition, eq 10, seems to be predominant at 500 °C, leading to a low CO_2_ formation. Likewise, the increase in the temperature clearly disfavors the methanation reaction, eq 12, or conversely, favors the inverse reaction, that is, methane steam reforming.

**Table 2:** Calculated Equilibrium Product Yields for the ESR at 1.4 bar, a Steam/Ethanol Molar Ratio of 3, and an Ethanol Partial Pressure of 0.05 bar

component	500 °C	600 °C
H_2_	0.424	0.676
CO	0.121	0.439
CO_2_	0.477	0.428
CH_4_	0.401	0.133

**Figure 3:**
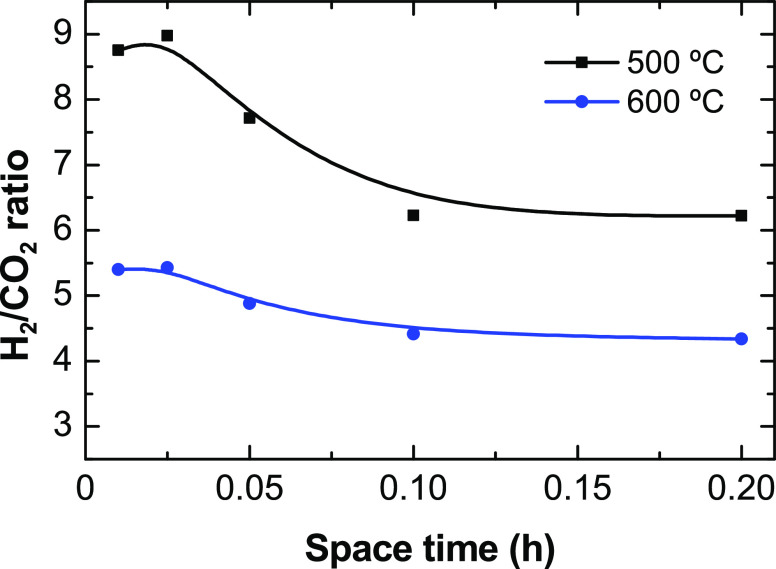
Evolution with space time of the H_2_/CO_2_ molar ratio in ESR at 500 and 600 °C.

To verify the relative relevance of H_2_ formation routes by decomposition of ethanol with respect to steam reforming reactions, we calculated the H_2_/CO_2_ molar ratio for all the experiments described in Figure 2. According to the stoichiometry of the reactions involved, a H_2_/CO_2_ ratio equal to 3 would indicate the favored extent of the steam reforming and water–gas shift reactions, whereas values above 3 would indicate that other H_2_ formation routes not forming CO_2_ are favored. The results (Figure 3) show that the H_2_/CO_2_ ratio decreases with increasing space-time values, and the values are much higher at 500 °C than at 600 °C, being above 3. Thus, increasing the space time and temperature favors steam reforming and water–gas shift reactions, albeit reaction routes for H_2_ formation by ethanol or ethylene decomposition are mostly predominant, particularly at 500 °C.

### 3.3 Role of Ethylene as an Intermediate

As expected, the aforementioned results reveal that ethylene is an important intermediate in the ESR on the Ni/Al_2_O_3_ catalyst used. Therefore, to confirm the relevance for this catalyst of the route of H_2_ and carbon formation in the ESR by the decomposition of the ethylene formed by ethanol dehydration, we carried out experiments using ethanol and ethylene as reactants at similar reaction conditions with different feeds. These experiments were carried out at a low space time (0.025 h), a condition with rapid deactivation. The results are analyzed in terms of the evolution with time on stream of the conversion and product yields at 500 and 600 °C with the purpose of determining the effect of catalyst deactivation on the routes for the H_2_ formation: (i) ethanol dehydration and ethylene decomposition and (ii) ethanol steam reforming and the water–gas shift reaction.

#### 3.3.1 Ethanol–Water Feed

**Figure 4:**
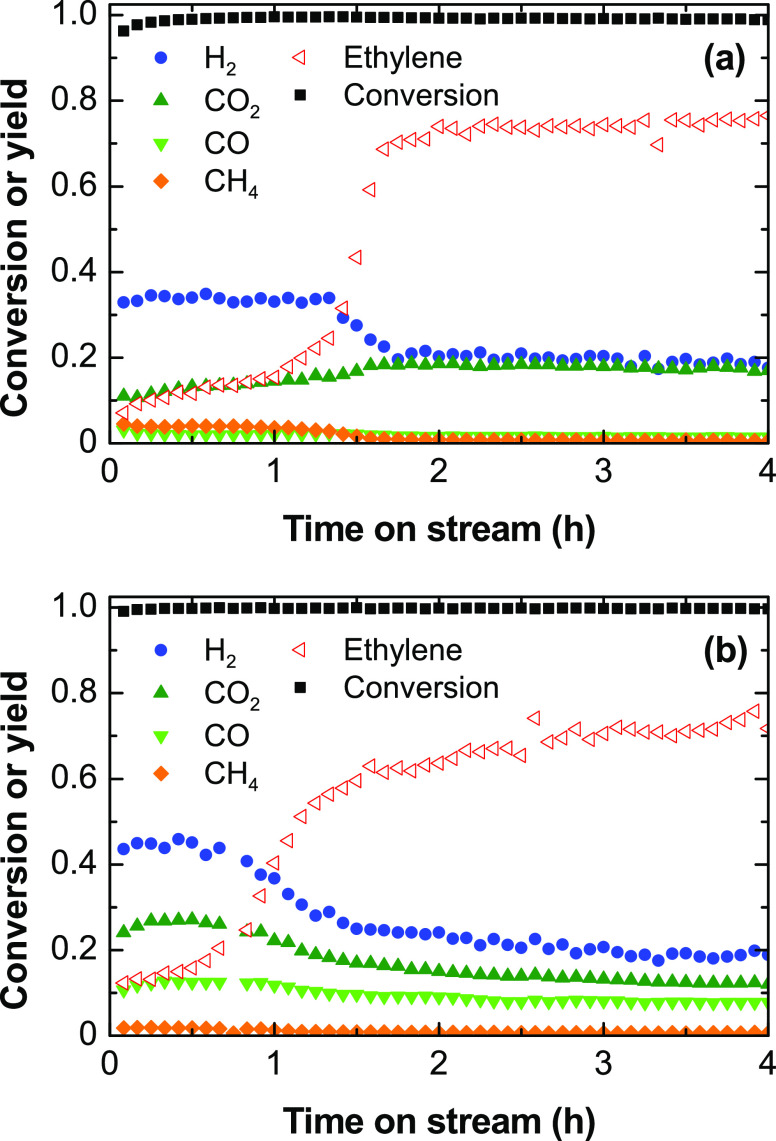
Evolution with time on stream of the ethanol conversion and product yields in ESR at (a) 500 and (b) 600 °C. Reaction conditions: space time, 0.025 h; ethanol partial pressure, 0.05 bar; steam/ethanol/N_2_ molar ratio, 3:1:16.

Figure 4 shows the evolution with time on stream of the ethanol conversion and product yields at 500 °C (Figure 4a) and 600 °C (Figure 4b). In general, the conversion is complete during 4 h at both temperature values, whereas the product distribution varies with time on stream. The H_2_ yield is high at the beginning of the run and then decreases after a certain time on stream, and simultaneously, the ethylene yield increases. This change in trend indicates a change in the prevailing H_2_ formation route, which is characteristic of parallel reactions that are selectively deactivated: at the beginning of the run, the catalyst is active for the H_2_ and carbon formation from ethylene, eq 10, and then it deactivates for this reaction and keeps active for steam reforming, eqs 1 and 9, and water–gas shift, eq 2, reactions.

**Figure 5:**
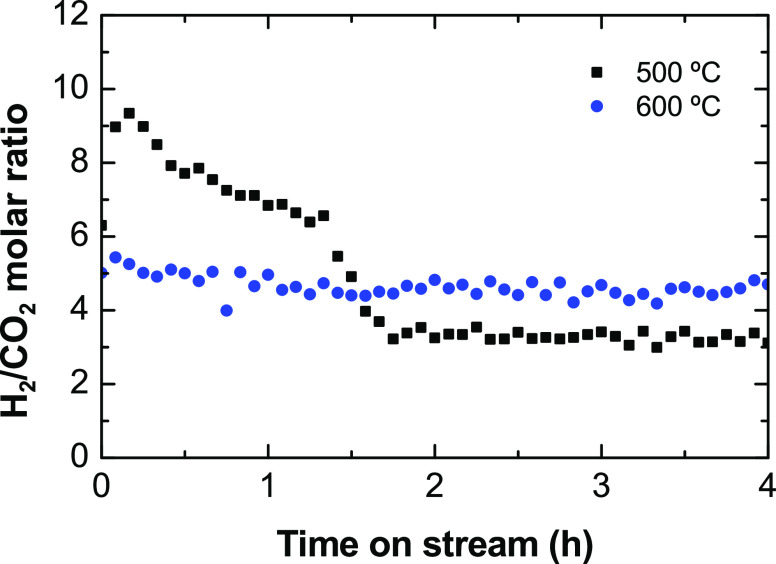
Evolution with time on stream of the H_2_/CO_2_ molar ratio in the ESR at a space time of 0.025 h, steam/ethanol ratio of 3, and at 500 or 600 °C (experiments described in Figure 4).

The evolution with time on stream of the H_2_/CO_2_ ratio at 500 °C (Figure 5) evidences the shift in the prevailing H_2_ formation routes, as previously discussed. H_2_/CO_2_ ratio values higher than 3 at the beginning of the run indicate the prevalent extent of the H_2_ formation from ethylene through eq 10, and values decreasing down to 3 indicate that the catalyst deactivation selectively decreases the rate for this reaction. Likewise, at 600 °C, the H_2_/CO_2_ ratio is slightly high at the beginning of the run and rapidly decreases being constant during the run. The value above 3 indicates that the extent of the global steam reforming reaction, eq 3, is not complete, possibly due to the thermodynamically disfavored water–gas shift reaction at this temperature. This also indicates that the extent of direct ESR route, eq 3, is not relevant on this catalyst, conversely to what is observed on a Ni/La_2_O_3_–αAl_2_O_3_ catalyst.^33^

For comparison, Figure S2 (Supporting Information) shows the conversion and yield of products (Figure S2a) and H_2_/CO_2_ ratio (Figure S2b) obtained with the Ni/Al_2_O_3_ catalyst prepared by impregnation (Section 2.1). The results have been obtained under the same conditions as in Figure 4a. As observed, the ethanol conversion is high, but it is not complete throughout the experiment. At the beginning of the experiment, there is a higher yield of carbon gaseous products and lower H_2_/CO_2_ molar ratio for the catalyst prepared by impregnation (Figure S2) compared to the values of the NiAl_2_O_4_ spinel derived catalyst (Figures 4a and 5). These results evidence significant differences in the prevailing H_2_ formation routes for both catalysts, in spite of their similar composition. The H_2_ formation route by ethanol dehydration followed by ethylene decomposition is less promoted with the Ni/Al_2_O_3_ catalyst prepared by impregnation than with the NiAl_2_O_4_ spinel derived catalyst, due to the lower acidity of the catalyst prepared by impregnation (Figure S1c). Interestingly, the continuous decreasing H_2_ yield from the beginning of the run evidence the lower stability of the catalyst prepared by impregnation.

#### 3.3.2 Pure Ethylene Feed

**Figure 6:**
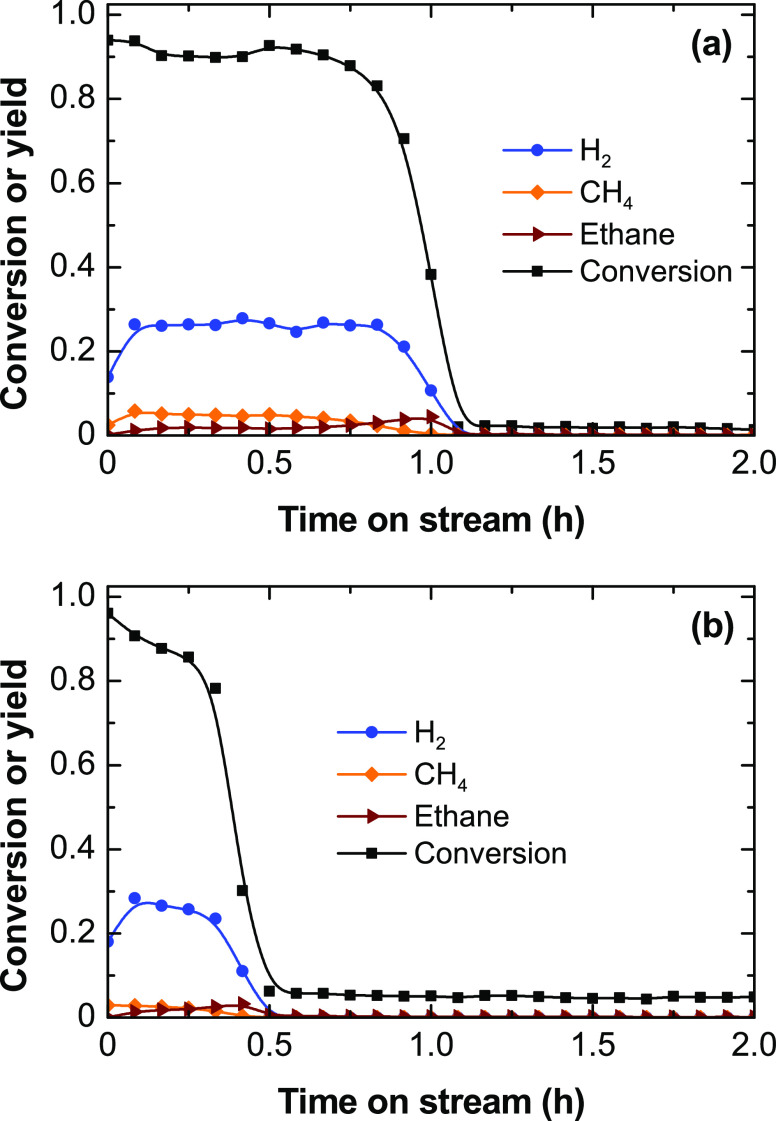
Evolution with time on stream of the ethylene conversion and product yields at (a) 500 and (b) 600 °C. Reaction conditions: space time, 0.041 h; ethylene partial pressure, 0.05 bar; steam/ethylene/N_2_ molar ratio, 0:1:19.

To confirm the relevance of the ethylene decomposition for H_2_ production, eq 10, on this catalyst, runs feeding pure ethylene (without steam) were carried out with a flow rate (1.22 g h^–1^ or 0.0435 mol h^–1^) equivalent to that formed from the complete ethanol dehydration at the conditions of Figure 4 (ethanol flow rate of 2 g h^–1^ or 0.0435 mol h^–1^). Therefore, the equivalent space time is 0.041 h when using the ethylene feed. Figure 6 shows the evolution with time on stream of the ethylene conversion and product yields at 500 °C (Figure 6a) and 600 °C (Figure 6b). The results show that H_2_ is the main gaseous product with traces of CH_4_ and ethane. The catalyst is very active at the beginning of the run, with ethylene conversion values above 90%, and deactivates rapidly, the deactivation being faster at 600 than at 500 °C (fully deactivated after 0.5 and 1 h on stream, respectively).

#### 3.3.3 Ethylene–Water Feed

**Figure 7:**
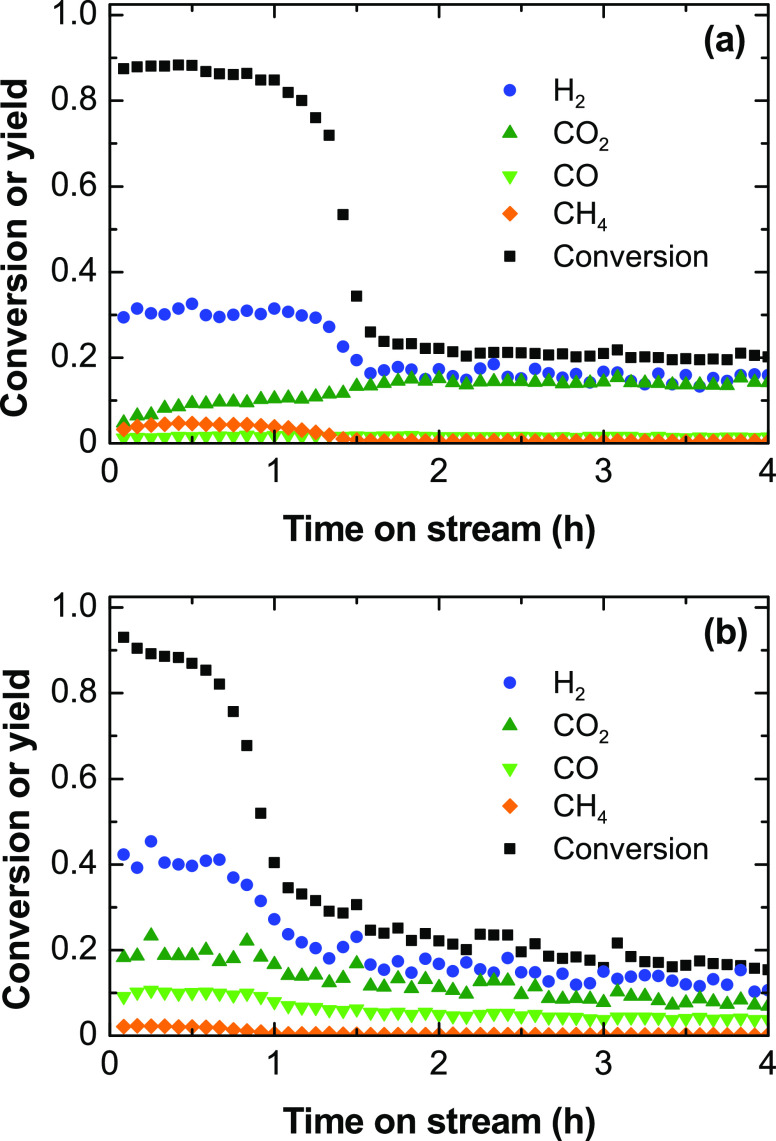
Evolution with time on stream of the ethylene conversion and product yields for the ethylene steam reforming at (a) 500 and (b) 600 °C. Reaction conditions: equivalent space time, 0.025 h; ethylene partial pressure, 0.05 bar; steam/ethylene/N_2_ molar ratio, 3:1:16.

Runs for the ethylene steam reforming reaction were carried out using a steam/ethylene ratio of 3 and the equivalent ethylene flow rate (1.22 g h^–1^) and space time (0.041 h) defined in the previous section for a pure ethylene feed. Figure 7 shows the evolution with time on stream of the ethylene conversion and product yields at 500 °C (Figure 7a) and 600 °C (Figure 7b). The product distribution at both temperature values is quite similar to that obtained for the ESR described in Figure 4 at 500 or 600 °C, with slightly lower yield values. The ethylene conversion decreases rapidly at certain time on stream in Figure 7 in a similar way to the increase in the ethylene yield described in Figure 4. These results confirm that ethylene is the main reaction intermediate in the ESR reaction and responsible for the H_2_ formation on the fresh catalyst. Ethylene is converted to H_2_ and carbon, eq 10, at the beginning of the run, and when the catalyst deactivates for this reaction, the prevailing reaction routes are ethylene steam reforming, eq 9, and the water–gas shift reaction, eq 2. The lower yields observed in these runs may be attributable to the absent contribution of the ESR reaction, eq 1, in this experiment.

#### 3.3.4 Pure Ethanol Feed

Finally, to verify the role of the ethanol decomposition, eq 6, runs feeding pure ethanol (without steam) were carried out at similar reaction conditions to those of the ESR (500 and 600 °C and space time of 0.025 h). Figure S3 shows the evolution with time on stream of the ethanol conversion and product yields at 500 °C (Figure S3a) and 600 °C (Figure S3b). The effect of temperature on the thermodynamics of the reactions may explain the differences in the results. At 500 °C and zero time on stream, ethanol undergoes dehydration to yield ethylene, eq 5, and ethylene undergoes mainly decomposition to yield H_2_, eq 10. After 60 min, the increase in the ethylene yield and complete ethanol conversion indicate that the catalyst deactivation selectively affects the ethylene conversion into H_2_ and carbon, eq 10, but the catalyst remains active for the ethanol dehydration reaction, eq 5. Furthermore, the low H_2_ and CO_2_ yields indicate that the catalyst also remains partially active for steam reforming, eqs 1 and 9, and the water–gas shift, eq 2, reactions that take place because of the presence of water formed from the ethanol dehydration. Curiously, the product yields provide no significant evidence of the ethanol thermal decomposition reaction, eq 6. At 600 °C, ethanol dehydration is limited by the thermodynamics with the consequent lower ethylene yield. Additionally, the presence of CO at zero time on stream evidences that steam reforming takes place with an incomplete water–gas shift reaction (also limited by thermodynamics). The catalyst partially deactivates with time on stream, leading to a progressive decrease in the ethanol conversion and ethylene yield, which indicates that the catalyst deactivates for the ethanol dehydration at these conditions (without water in the feed). The increase in the acetaldehyde yield with time on stream indicates that ethanol dehydrogenation, eq 4, is also relevant at the end of the run, contributing to the residual H_2_ yield at high time on stream when the catalyst is deactivated for other routes. Thus, the ethanol dehydrogenation seems to be relevant in the absence or with low concentrations of water. It is noteworthy to mention that the presence of acetaldehyde as an intermediate undergoing fast decomposition, eq 7, and steam reforming, eq 8, reactions is a common reaction route reported for the ESR on less acidic catalysts.^11,32^

### 3.4 Catalyst Stability in Long-Duration Experiments

**Figure 8:**
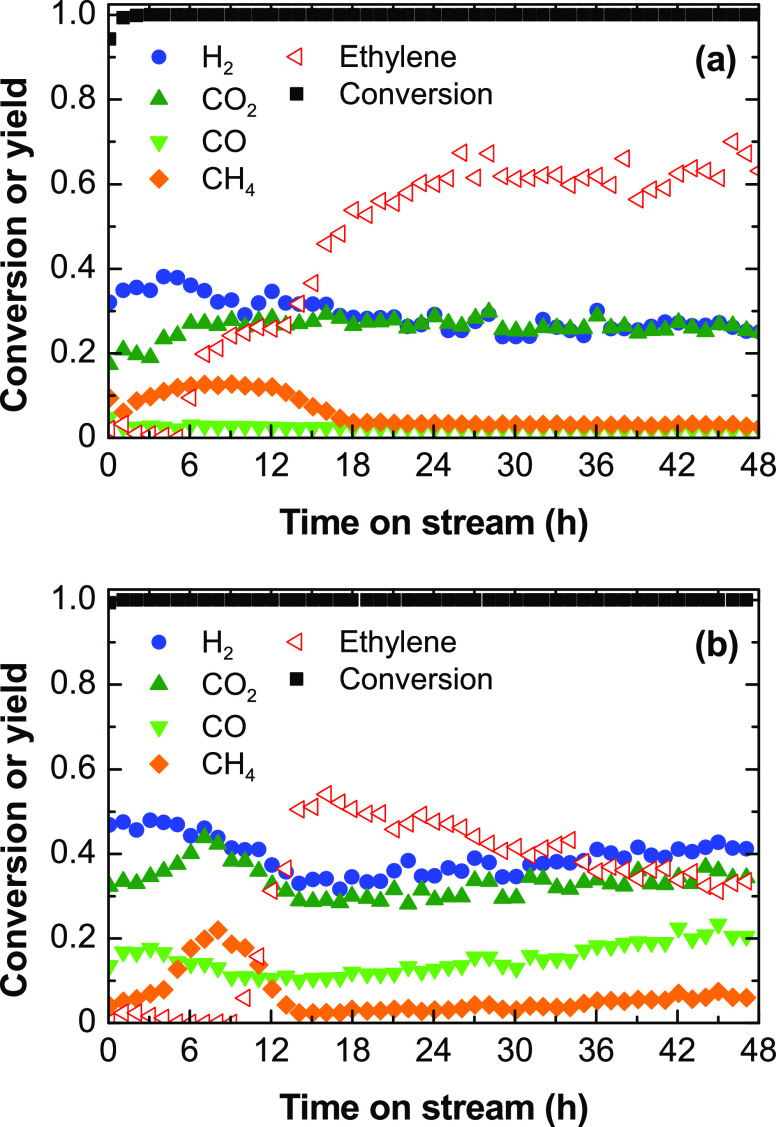
Evolution with time on stream of the ethanol conversion and product yields in ESR at (a) 500 and (b) 600 °C. Reaction conditions: space time, 0.1 h, ethanol partial pressure, 0.05 bar, steam/ethanol/N_2_ molar ratio, 3:1:16.

Figure 8 shows the evolution with time on stream of the ethanol conversion and gaseous product yields at 500 °C (Figure 8a) and 600 °C (Figure 8b) in long-duration experiments, whereas Figure S4a shows the corresponding evolution of solid carbon yield with time on stream. For these experiments, the space time is set at 0.1 h in order to obtain the maximum H_2_ yield according to the results described in Figure 2. The conversion is complete and remains constant during 48 h on stream at both temperature values, however, with notorious variations in the product distribution along an initial transient period of 23 h at 500 °C and 15 h at 600 °C. After this transient period, the catalyst acquires a pseudostable behavior, without appreciable variations with time on stream in the distribution of products at 500 °C (Figure 8a) and with small variations at 600 °C (Figure 8b). In the pseudostable state of the catalyst, a remarkable yield of H_2_ and ethylene as gaseous products of interest remains constant. This indicates that the catalyst keeps a high activity for the ethanol dehydration, eq 5, and for the H_2_ formation routes (steam reforming, eqs 1 and 9, and water–gas shift, eq 2, reactions).

The analysis of the product distribution evolution in the transient period in Figures 8 and S4a provides further insights into the H_2_ formation mechanisms and catalyst deactivation, giving evidence that the catalyst deactivates selectively for the different reactions in the kinetic scheme. At both temperature values, the evolution with time on stream of the product yields can be divided into different stages corresponding to different states of catalyst deactivation. In general, the H_2_ yield is high and constant in the first 5 h on stream and slight decreases afterward. Likewise, the ethylene yield is negligible at the beginning of the runs and increases rapidly after 6.3 h at 500 °C and 8.3 h at 600 °C, indicating the catalyst deactivation for the ethylene conversion through eq 10, which is in agreement with the decrease in solid carbon yield at the beginning of the reaction (Figure S4a). The CO yield evolves almost in parallel to the H_2_ yield at 600 °C, indicating that both H_2_ and CO are formed through the ESR reaction, eq 1. The increase in the CO_2_ and CH_4_ yields with time on stream at the beginning of the reaction indicates that, as the catalyst deactivates for the ethylene decomposition reaction, the steam reforming of ethylene (eq 9) and, consequently, the water–gas shift (eq 2) and methanation (eq 12) reactions, are favored. The maximum in the CH_4_ yield indicates that the catalyst deactivation subsequently affects the methanation reaction, eq 12. The maximum in the CO_2_ yield at 600 °C may be related to the slight decrease observed in the CO concentration and the partial deactivation of the catalyst for the water–gas shift reaction at this temperature. At 600 °C, the slow decrease in ethylene yield with the time on stream after its maximum value at 15 h on stream shows the progressive deactivation of the catalyst for the dehydration of ethanol (Figure 8b). However, after this time on stream, the ethanol conversion remains constant and complete, and the yield of H_2_, CO, and CO_2_ increases, because the catalyst maintains a remarkable activity for ethanol steam reforming and water–gas shift reactions.

These results make evident that the H_2_ yield and selectivity (related to the concentration in the gaseous product stream) is high when the ethylene conversion to H_2_ and carbon prevails, particularly at low temperature. Figure S4b shows the evolution with time on stream of the H_2_ selectivity for the experiments described in Figure 8. The experimental values are compared with those corresponding to thermodynamic equilibrium calculations (Table 2). The comparison shows that the experimental H_2_ selectivity is higher than that predicted through the thermodynamics at 500 °C in the period when the ethylene conversion to H_2_ and carbon remains active, and decreases down to a value similar to the thermodynamic H_2_ selectivity when the catalyst deactivates for this reaction (as evidenced by the decrease in carbon yield observed in Figure S4a and the increase in ethylene yield in Figure 8a)

### 3.5 Carbon Product: Quantification, Characteristics, and Role in Deactivation

**Figure 9:**
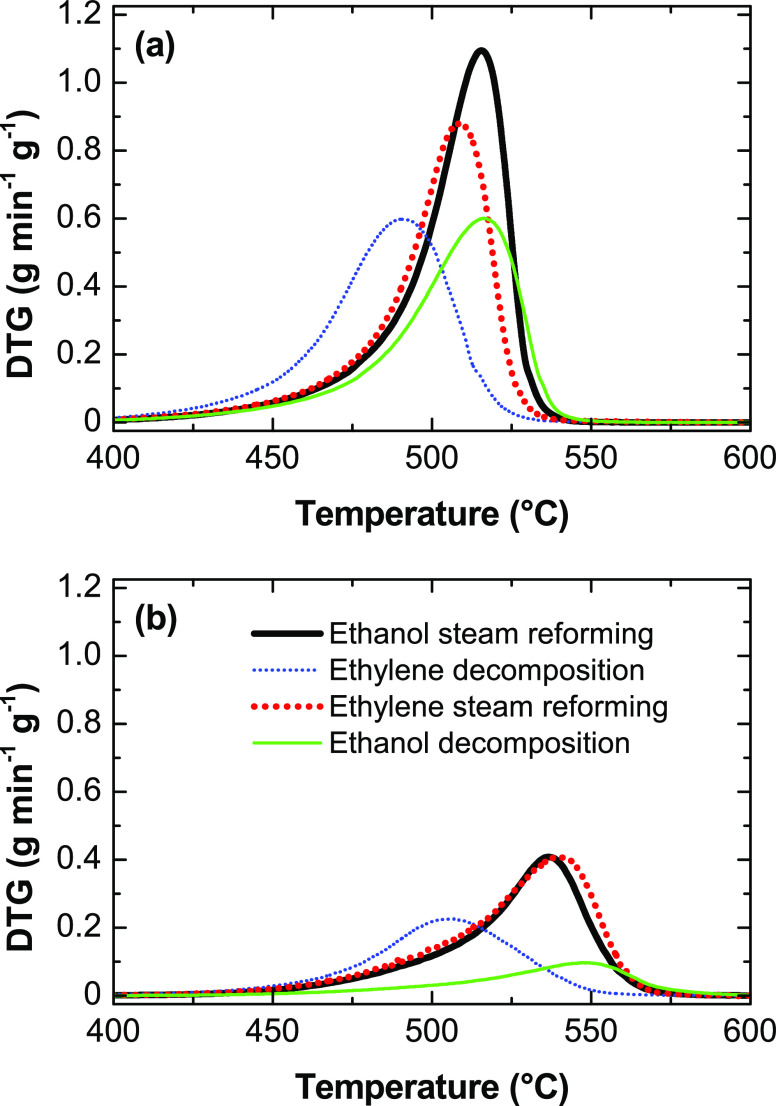
TPO profiles of carbon formed in the experiments with different feeds at (a) 500 and (b) 600 °.C Reaction conditions: space time, 0.025 h, time on stream, 4 h (corresponding to the results described in Figures 4, 6, 7, and S3).

**Figure 10:**
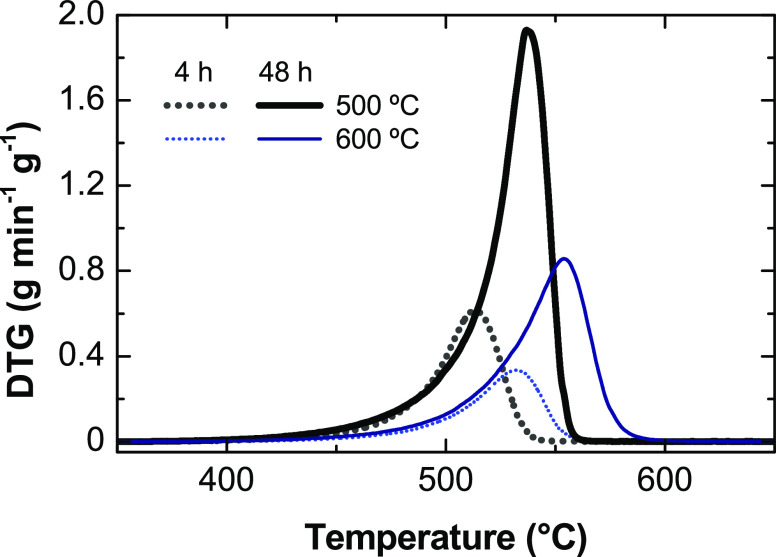
TPO profiles of carbon formed in the ESR at 500 and 600 °C with experiment durations of 4 and 48 h on stream (corresponding to the results described in Figure 8).

The carbon material is abundantly formed through ethylene conversion, eq 10, and CO disproportionation, eq 11. As aforementioned, the deposition of carbon on the catalyst surface contributes to catalyst deactivation, but the carbon formation in large amounts and with specific characteristics also offers a commercial interest. For quantifying and characterizing the carbon material, TPO analyses were carried out as described in Section 2.2. The TPO profiles shown in Figure S5 correspond to the carbon material formed in the experiments described in Figure 2. Figures 9 and 10 show the TPO profiles corresponding to the carbon material formed in the conditions of Figures 4 and 6–8. The TPO profiles essentially show a unique combustion peak centered at different temperature values according to the reaction conditions. Nevertheless, the position of the maximum of the combustion peak is not affected by the amount of coke deposited (corresponding to the area under each TPO profile), as evidenced by the similar position of the maximum for the different values of space time at 500 °C (Figures S5a) and 600 °C (Figure S5b). Thus, the combustion peaks of carbon formed in the ESR experiments at different space-time values and with a duration of 4 h on stream (Figure S5) are centered around 514 °C for the experiments at 500 °C and around 538 °C for the experiments at 600 °C. Likewise, the TPO profiles corresponding to different feeds (Figure 9) evidence that carbon formed in the conversion of pure ethylene at 500 and 600 °C burns at lower temperature (491 and 506 °C, respectively) than carbon formed from other feeds. Thus, for ethylene steam reforming, these peaks are centered at 509 and 539 °C, for ethanol steam reforming, they are centered at 515 and 537 °C, and for ethanol decomposition, they are centered at 516 and 547 °C for the experiments at 500 and 600 °C, respectively. The combustion peaks of carbon formed in the ESR experiments with different duration (Figure 10) are centered at 513 °C for 4 h and 540 °C for 48 h at 500 °C and at 532 °C for 4 h and 554 °C for 48 h at 600 °C. As previously commented, these differences in the temperature of the maximum for different duration should not be attributed to differences in the amount of coke deposited but to differences in the carbon structure.

Therefore, the analysis of the TPO profiles proves that (i) the carbon formed is more refractory with the increase in the temperature and in the duration of the run as a consequence of its structure development (aging); (ii) the carbon formed in the ethylene conversion through eq 10 is less refractory than in the ethanol decomposition and ethanol or ethylene steam reforming, most likely because the Boudouard reaction (which results in a more condensed carbon) does not contribute to its formation due to the absence of CO in the reaction medium. It should be noted that there is a direct relationship between the concentration of CO in the reaction medium and the temperature of the maximum of the combustion peak.

The amount of carbon formed (with respect to the catalyst weight) is significantly high for all the experiments, giving evidence of the prevailing extent of the carbon formation routes on the catalyst used in this work. Figure S6 shows the evolution with space time of the amount of carbon formed in 4 h for the experiments described in Figure 2. The carbon amount shows a maximum value for a space time of 0.05 h at both temperature values and is remarkably higher at 500 than at 600 °C. These high values confirm the dominant ethylene decomposition route yielding H_2_ and carbon, eq 10, without ruling out the formation through the CO disproportionation, eq 11, whose extents are more favored at 500 than at 600 °C. Likewise, the lower amount at 600 °C is also attributable to the favored extent of the gasification reactions, eq 13, and the reverse of eq 11 at this temperature. The favored extent of the route of ethylene conversion to H_2_ and carbon deposits would explain the low yields of gaseous carbon products (CO, CO_2_, and CH_4_) in comparison to the expected equilibrium yields (Table 2).

Table 3 summarizes the carbon amount and average carbon formation rate for the different reaction type experiments described in Figures 4, 6, 7, and S3. The amount is generally higher at 500 than at 600 °C, again indicating the effect of the increase in the temperature on favoring gasification reactions and disfavoring the CO disproportionation reaction.

**Table 3:** Carbon Amount (g g^–1^) and Carbon Formation Rate (g g^–1^ h^–1^) for Different Reactions and Temperature Values at a Space Time of 0.025 h (Duration = 4 h)

	carbon amount (g g^–1^)		carbon formation rate (g g^–1^ h^–1^)	
feed	500 °C	600 °C	500 °C	600 °C
ethanol–water	18.7	9.32	4.67	2.33
pure ethanol	13.3	2.93	3.32	0.732
ethylene–water	15.9	10.4	3.97	2.60
pure ethylene	14.5	6.52	3.62	1.63

**Figure 11:**
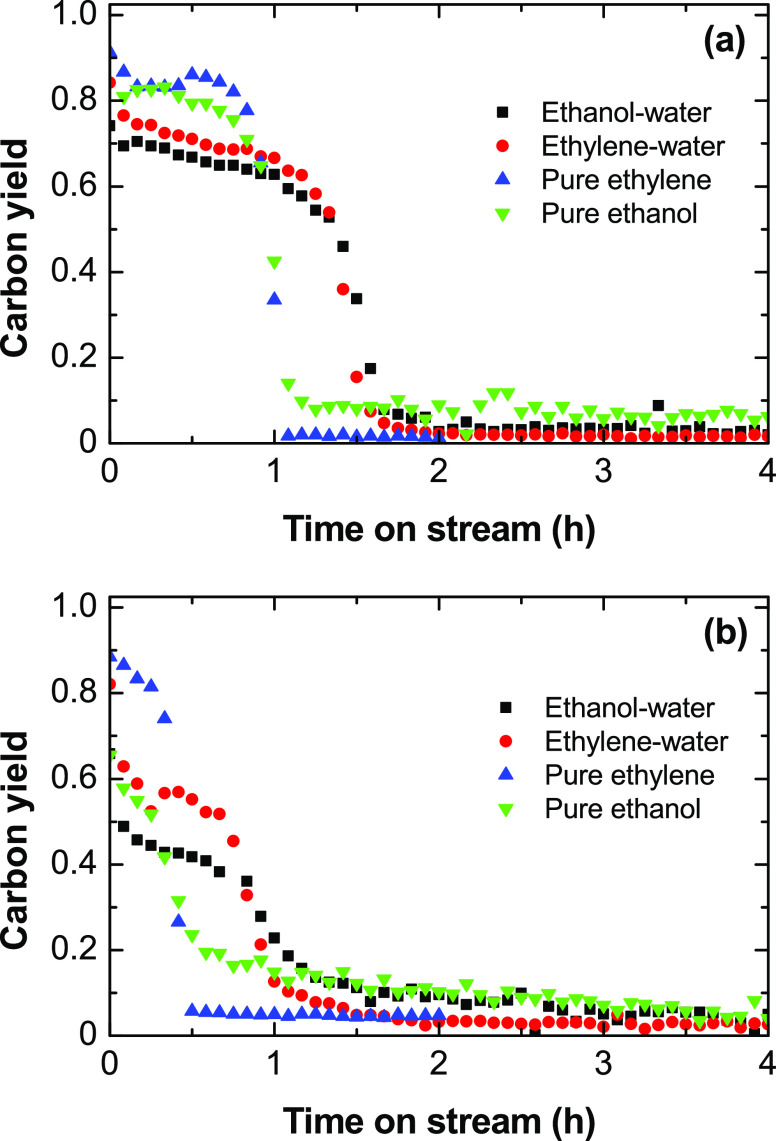
Evolution with time on stream of solid carbon yield estimated from carbon balance for the feeds of ethanol–water, ethylene–water, pure ethanol, or ethylene at (a) 500 and (b) 600 °C.

Interestingly, the carbon amount deposited is higher in the runs with water in the feed than without water, which can be explained by relevance of deactivation in the mechanism of carbon formation/gasification. Thus, carbon material is mainly formed by the ethylene decomposition reaction, eq 10, and also, it is converted into other products (CO in the gas) by the reverse Boudouard reaction, eq 11, or gasification with water, eq 13. Consequently, the formation of carbon depends on the ethylene concentration and the extent of gasification reactions, which are in turn dependent on space time (catalyzed reactions), temperature, water content, and the activity of the catalyst for gasification. For a better interpretation of the results of Table 3, Figure 11 shows the evolution with time on stream of the solid carbon yield (determined by carbon balance throughout the experiment) for the different feeds in Table 3. It is observed that the carbon yield is maximized at the beginning of the reaction and decreases with time on stream, with an abrupt decrease after a certain time, in parallel to the rapid increase in the ethylene yield observed in Figures 4, 6, 7, and S3. This result is due to the deactivation of the catalyst for the ethylene decomposition reaction, eq 10. When water is cofed, the initial carbon yield is lower (due to the greater extent of the gasification reaction, eq 13), but the deactivation rate of the catalyst for the carbon formation reaction is slower. Consequently, the carbon yield decreases slower with time on stream, which explains the higher carbon content after 4 h of reaction as shown in Table 3 for reactions cofeeding water (S/E = 3).

Table 4 summarizes the carbon amount and average carbon formation rate in the ESR experiments with different durations and a high value of space time (0.1 h). Interestingly, the carbon formation is faster at the beginning of the run, giving an average formation rate of 3.05 g g^–1^ h^–1^ at 500 °C and 1.84 g g^–1^ h^–1^ at 600 °C for a period of 4 h in comparison to 0.704 g g^–1^ h^–1^ at 500 °C or 0.417 g g^–1^ h^–1^ at 600 °C for 48 h. This may be indicative of the prevailing extent of the ethylene decomposition at the beginning of the run, leading to a high carbon formation. After the initial transient period, the steam reforming and water–gas shift are the prevailing reactions, leading to a low carbon yield (as shown in Figure S4a).

**Table 4:** Carbon Amount (g g^–1^) and Average Carbon Formation Rate (g g^–1^ h^–1^) for the Experiments Described in Figure 10 for 4 and 48 h Durations (Space Time of 0.1 h)

	carbon amount (g g^–1^)		carbon formation rate (g g^–1^ h^–1^)	
duration	500 °C	600 °C	500 °C	600 °C
4 h	12.2	7.36	3.05	1.84
48 h	33.8	20.0	0.704	0.417

**Figure 12:**
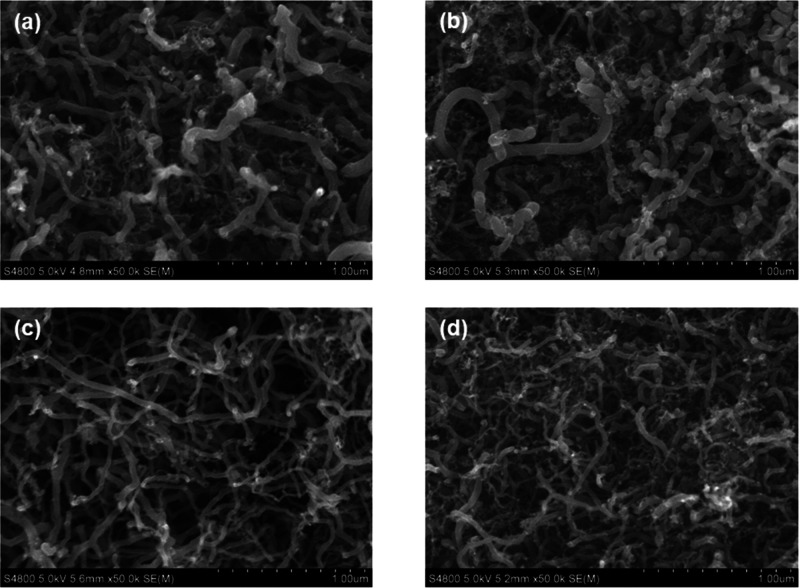
SEM images of carbon formed in the ESR at 500 °C for (a) 4 h and (b) 48 h and at 600 °C for (c) 4 h and (d) 48 h. Space time of 0.1 h.

**Figure 13:**
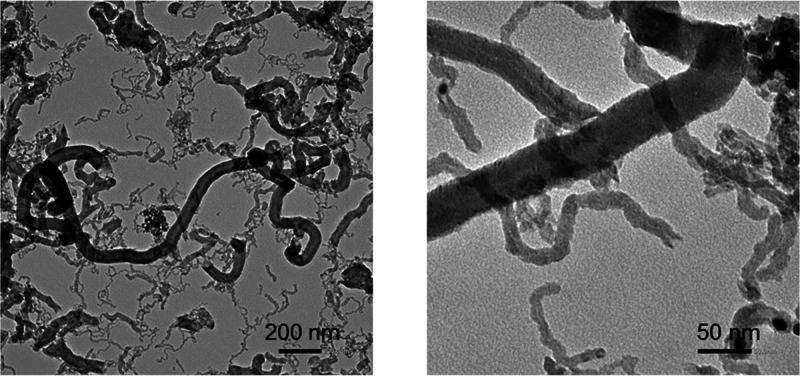
Two TEM images of carbon formed in the ESR after 48 h. Reaction conditions: 500 °C, space time, 0.1 h.

The SEM and TEM analyses are conclusive for determining the morphology of the carbon material formed. Figure 12 shows the SEM images of this material after 4 and 48 h for the experiments described in Figure 8. The morphology corresponds to carbon filaments for both experiments. These filaments are heterogeneous in size, being similar regardless of the experiment duration (4 or 48 h) and slightly larger in diameter at 500 than at 600 °C. Likewise, the TEM analysis of the catalyst used at 500 °C during 48 h (Figure 13) reveals that the carbon filaments are hollow, indicating the formation of carbon nanotubes. Additionally, these images show dark particles corresponding to Ni crystals along the carbon nanotubes. The presence of Ni crystals suggests that the carbon nanotubes are formed through the well established tip-growth mechanism,^20^ which makes part of the Ni surface accessible for reactants (Ni crystals supported on a carbon structure). However, Ni crystals may be partially trapped in the interior of the nanotubes, reducing the Ni surface available for reactions, which leads to a partial catalyst deactivation. Apparently, based on the aforementioned results, the formation of carbon nanotubes has low impact on the activity of the Al_2_O_3_ acid sites, since the ethanol dehydration reaction maintains a noticeable extent over the time on stream in the ESR experiments at 500 °C (Figure 8a), although there is a show and progressive deactivation of the catalyst for this reaction at 600 °C (Figure 8b).

### 3.6 Remarks on the Reaction Routes

**Figure 14:**
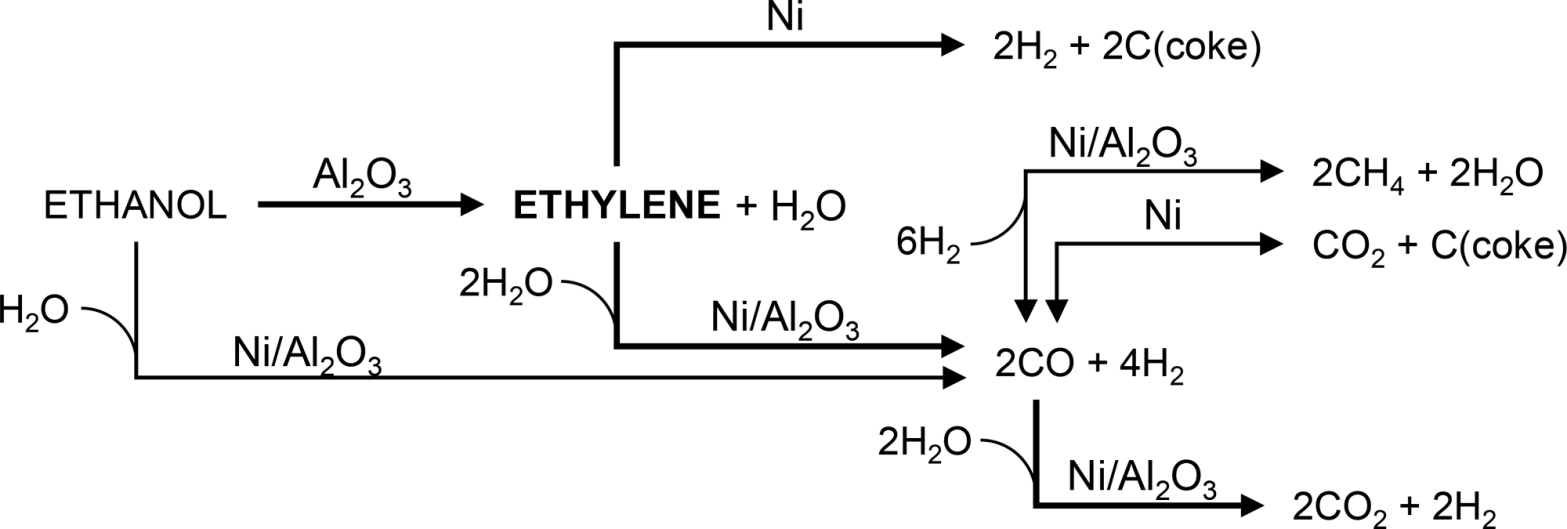
Main reaction routes in the ESR on the Ni/Al_2_O_3_ catalyst derived from NiAl_2_O_4_ spinel in the 500–600 °C range.

Upon analyzing the gaseous and solid products (Sections 3.3 and 3.5, respectively) obtained at different conditions in the ethanol and ethylene steam reforming reactions and in the ethylene and ethanol decomposition reactions, the role of ethylene as reaction intermediate with this catalyst is verified for the formation of H_2_ and carbon nanotubes in the ESR. Thus, the presence of ethylene in the product stream is a result of the insufficient space time for the complete conversion of ethylene or of catalyst deactivation (that selectively affects to this route). Hence, based on this, Figure 14 pictures the main reaction routes taking place in the ESR on the Ni/Al_2_O_3_ catalyst derived from NiAl_2_O_4_ spinel, in the conditions used in this work.

This reaction network implies a series-parallel pathway. The ethanol dehydration, eq 5, takes place on the Al_2_O_3_ acid sites (support) forming ethylene and water. Ethylene reacts on the interface Ni–Al_2_O_3_ yielding H_2_ and carbon nanotubes, eq 10, whereas the Ni sites catalyze the ethylene steam reforming, eq 9, yielding H_2_ and CO. The ESR reaction, eq 1, also takes place on the Ni sites yielding CO and H_2_. Likewise, the Ni sites catalyze the water–gas shift reaction, eq 2, converting CO into CO_2_ and H_2_, and the methanation reaction, eq 12, converting CO into CH_4_. Additionally, Setiabudi el at.^59^ suggest that the role of the Al_2_O_3_ support is to adsorb water and form −OH species that react with the reactive species adsorbed on Ni sites. Also, the CO disproportionation (Boudouard reaction, eq 11) may take place on Ni sites contributing to form carbon and CO_2_. The extent of the conversion of CO through the water–gas shift, methanation, or disproportionation reactions depends on the temperature, being highly favored at 500 °C and partially disfavored at 600 °C. The carbon gasification reaction, eq 13 (not considered in this scheme), also takes place, especially at high temperature values, decreasing the net carbon formation. Other minor routes (not included in Figure 14) also take place such as the ethanol dehydrogenation, eq 4, yielding acetaldehyde and the subsequent acetaldehyde decomposition, eq 7, and steam reforming, eq 8.

The catalyst selectively deactivates for the formation of H_2_ and carbon nanotubes from ethylene, eq 10, and the methanation reaction (eq 12) and keeps active for ethanol dehydration (eq 5) and steam reforming (eqs 1, 3,7, and 9) and the water–gas shift (eq 2) reactions. The selective catalyst deactivation can be explained by a transition on the state of Ni sites on the catalyst structure. Initially, the high carbon formation through the ethylene decomposition, eq 10, on the Ni-support sites causes the detachment of Ni crystals from the support and suppresses the necessary Ni-support interaction for this reaction, thus causing its deactivation. Subsequently, in the carbon growth process, some Ni crystals may be trapped in the carbon nanotubes suppressing the methanation reaction, but most of the Ni sites would be located on the tip of carbon nanotubes, being accessible to the reactants and providing a high remaining activity for steam reforming and water–gas shift reactions, whereas the Al_2_O_3_ acid sites would remain active for ethanol dehydration.

In the literature, the presence of acid sites in the support is considered a problem, because these sites activate the dehydration of ethanol that is an undesired route, because ethylene is responsible for the formation of a carbonaceous material (coke) that deactivates the catalyst.^5,13^ Thus, the common strategy to control ethylene formation and subsequent deactivation by carbon deposition is the support modification to neutralize the acid sites.^18^ However, taking into account the aforementioned results, with the catalyst used in this work and in a fluidized-bed reactor, the deactivation of the catalyst by carbon formation is only a transitory problem, which selectively affects the formation of carbon itself (mainly by decomposition of ethylene). Consequently, this carbon formation, with the morphology of nanotubes, can be considered as an opportunity to boost the economy for the H_2_ production.^20^ The use of low values of temperature (500 °C) would be beneficial for increasing the formation of carbon nanotubes with a high H_2_ selectivity (Figure S4b). The favored route of ethanol dehydration followed by ethylene decomposition at certain reaction conditions on this catalyst compared to the Ni/Al_2_O_3_ catalyst prepared through wet impregnation (as evidenced by the comparison of Figures 4a and S2) would yield a gaseous product stream rich in H_2_, with lower CO_2_ formation, because part of the carbon is converted into valuable carbon nanotubes. If the process were targeted for the coproduction of H_2_ and carbon nanotubes, it would be interesting to operate for short time on stream, when the carbon yield is maximum (Figures 11 and S4a).

## 4 Conclusions

The results of this work bring to light the reaction routes prevailing in the ethanol steam reforming at moderate temperature (500–600 °C) on a Ni/Al_2_O_3_ catalyst derived from NiAl_2_O_4_ spinel, which is a useful information for further studying ways to optimize the reaction conditions. Upon reducing NiAl_2_O_4_ spinel at 850 °C, the resulting Al_2_O_3_ phase contains acid sites that mainly catalyze the ethanol dehydration yielding ethylene as the main intermediate. Ethanol and ethylene react on Ni sites to yield H_2_, CO, CO_2_, and carbon material through decomposition, steam reforming, and water–gas shift reactions in a complex series-parallel reaction scheme. The extent of the ethylene decomposition forming H_2_ and carbon prevails at the beginning of the runs and is favored at low temperature (500 °C). The catalyst deactivates rapidly for this reaction but remains active for steam reforming and water–gas shift reactions, reaching a pseudostable state with high activity, H_2_ selectivity, and stability. The increase in the space time and temperature favors the extent of the steam reforming reactions, whereas the extent of the water–gas shift reaction is partially suppressed at 600 °C because of its exothermic nature.

The carbon formation is partially attenuated at 600 °C due to the disfavored extent of the CO disproportionation reaction and, at the same time, the favored extent of the coke gasification reactions. The morphology of this material corresponds to carbon nanotubes, forming a tangle of carbon hollow fibers that make a porous structure letting reactants and products to diffuse and reach catalytic sites, which explains the high stability of the catalyst for converting ethanol. However, part of the Ni crystals may be trapped in the carbon nanotubes causing a partial catalyst deactivation for some reactions.

Overall, the performance of the catalyst used in this work is quite satisfactory for the ethanol steam reforming at 500 and 600 °C. It opens up opportunities for the coproduction of H_2_ and carbon nanotubes when the ethylene decomposition route prevails, particularly at 500 °C. This is an interesting result, because it contributes to the production of a high purity H_2_ stream with low CO_2_ emissions (due to the carbon sequestration in the form of carbon nanotubes).

The results are of interest to progress in the knowledge of the capacity of NiAl_2_O_4_ spinel to obtain a catalyst with high activity, selectivity, and stability in the ESR. Understanding the stages of the reaction pathway will help to improve catalyst properties and to set optimal conditions for H_2_ production. In this sense, the use of a fluidized-bed reactor has facilitated the experimental study, by maintaining an isothermal regime and allowing to run long-duration experiments with high carbon content, and the conditions of this reactor are also interesting for the scaling of the process.

## Supporting Information Available

Characterization and ESR reaction test data for the Ni/Al_2_O_3_ catalyst prepared through wet impregnation, data for the ethanol decomposition reaction and long-duration ESR experiments on the Ni/Al_2_O_3_ catalyst obtained from NiAl_2_O_4_ spinel, TPO and total carbon amount for ESR reaction experiments at different space-time values (PDF)

## References

[ref1] Abdin Z.; Zafaranloo A.; Rafiee A.; Mérida W.; Lipiński W.; Khalilpour K. R. Hydrogen as an Energy Vector. Renewable Sustainable Energy Rev. 2020, 120, 109620 10.1016/j.rser.2019.109620.

[ref2] Lamichhane G.; Acharya A.; Poudel D. K.; Aryal B.; Gyawali N.; Niraula P.; Phuyal S. R.; Budhathoki P.; Bk G.; Parajuli N. Recent Advances in Bioethanol Production from Lignocellulosic Biomass. Int. J. Green Energy 2021, 18 (7), 731–744. 10.1080/15435075.2021.1880910.

[ref3] Cordero-Lanzac T.; Aguayo A. T.; Gayubo A. G.; Bilbao J. Influence of HZSM-5-Based Catalyst Deactivation on the Performance of Different Reactor Configurations for the Conversion of Bioethanol into Hydrocarbons. Fuel 2021, 302, 121061 10.1016/j.fuel.2021.121061.

[ref4] Mattos L. V.; Jacobs G.; Davis B. H.; Noronha F. B. Production of Hydrogen from Ethanol: Review of Reaction Mechanism and Catalyst Deactivation. Chem. Rev. 2012, 112 (7), 4094–4123. 10.1021/cr2000114. 22617111

[ref5] Nanda S.; Rana R.; Zheng Y.; Kozinski J. A.; Dalai A. K. Insights on Pathways for Hydrogen Generation from Ethanol. Sustain. Energy Fuels 2017, 1 (6), 1232–1245. 10.1039/C7SE00212B.

[ref6] Landa L.; Remiro A.; de la Torre R.; Aguado R.; Bilbao J.; Gayubo G. Global Vision from the Thermodynamics of the Effect of the Bio-Oil Composition and the Reforming Strategies in the H_2_ Production and the Energy Requirement. Energy Convers. Manage. 2021, 239, 114181 10.1016/j.enconman.2021.114181.

[ref7] Montero C.; Oar-Arteta L.; Remiro A.; Arandia A.; Bilbao J.; Gayubo A. G. Thermodynamic Comparison between Bio-Oil and Ethanol Steam Reforming. Int. J. Hydrogen Energy 2015, 40 (46), 15963–15971. 10.1016/j.ijhydene.2015.09.125.

[ref8] Srisiriwat N.; Wutthithanyawat C. Autothermal Reforming of Ethanol for Hydrogen Production: Thermodynamic Analysis. Appl. Mech. Mater. 2013, 415 (I), 658–665. 10.4028/www.scientific.net/AMM.415.658.

[ref9] Lima da Silva A.; Malfatti C. de F.; Müller I. L. Thermodynamic Analysis of Ethanol Steam Reforming Using Gibbs Energy Minimization Method: A Detailed Study of the Conditions of Carbon Deposition. Int. J. Hydrogen Energy 2009, 34 (10), 4321–4330. 10.1016/j.ijhydene.2009.03.029.

[ref10] Montero C.; Remiro A.; Valle B.; Oar-Arteta L.; Bilbao J.; Gayubo A. G. Origin and Nature of Coke in Ethanol Steam Reforming and Its Role in Deactivation of Ni/La_2_O_3_-αAl_2_O_3_ Catalyst. Ind. Eng. Chem. Res. 2019, 58 (32), 14736–14751. 10.1021/acs.iecr.9b02880.

[ref11] Vicente J.; Ereña J.; Montero C.; Azkoiti M. J.; Bilbao J.; Gayubo A. G. Reaction Pathway for Ethanol Steam Reforming on a Ni/SiO_2_ Catalyst Including Coke Formation. Int. J. Hydrogen Energy 2014, 39 (33), 18820–18834. 10.1016/j.ijhydene.2014.09.073.

[ref12] Vizcaíno A. J.; Arena P.; Baronetti G.; Carrero A.; Calles J. A.; Laborde M. A.; Amadeo N. Ethanol Steam Reforming on Ni/Al_2_O_3_ Catalysts: Effect of Mg Addition. Int. J. Hydrogen Energy 2008, 33 (13), 3489–3492. 10.1016/j.ijhydene.2007.12.012.

[ref13] Muroyama H.; Nakase R.; Matsui T.; Eguchi K. Ethanol Steam Reforming over Ni-Based Spinel Oxide. Int. J. Hydrogen Energy 2010, 35 (4), 1575–1581. 10.1016/j.ijhydene.2009.12.083.

[ref14] Ogo S.; Sekine Y. Recent Progress in Ethanol Steam Reforming Using Non-Noble Transition Metal Catalysts: A Review. Fuel Process. Technol. 2020, 199, 106238 10.1016/j.fuproc.2019.106238.

[ref15] Jiménez-González C.; Boukha Z.; De Rivas B.; González-Velasco J. R.; Gutiérrez-Ortiz J. I.; López-Fonseca R. Behavior of Coprecipitated NiAl_2_O_4_/Al_2_O_3_ Catalysts for Low-Temperature Methane Steam Reforming. Energy Fuels 2014, 28 (11), 7109–7121. 10.1021/ef501612y.

[ref16] Contreras J. L.; Salmones J.; Colín-Luna J. A.; Nuño L.; Quintana B.; Córdova I.; Zeifert B.; Tapia C.; Fuentes G. A. Catalysts for H_2_ Production Using the Ethanol Steam Reforming (a Review). Int. J. Hydrogen Energy 2014, 39 (33), 18835–18853. 10.1016/j.ijhydene.2014.08.072.

[ref17] Gayubo A. G.; Vicente J.; Ereña J.; Montero C.; Olazar M.; Bilbao J. Comparison of Ni and Co Catalysts for Ethanol Steam Reforming in a Fluidized Bed Reactor. Catal. Lett. 2014, 144 (7), 1134–1143. 10.1007/s10562-014-1265-x.

[ref18] Sharma Y. C.; Kumar A.; Prasad R.; Upadhyay S. N. Ethanol Steam Reforming for Hydrogen Production: Latest and Effective Catalyst Modification Strategies to Minimize Carbonaceous Deactivation. Renewable Sustainable Energy Rev. 2017, 74, 89–103. 10.1016/j.rser.2017.02.049.

[ref19] Tian H.; Pei C.; Wu Y.; Chen S.; Zhao Z.; Gong J. Tunable Metal-Oxide Interaction with Balanced Ni^0^/Ni^2+^ Sites of Ni _x_ Mg_1–x_ O for Ethanol Steam Reforming. Appl. Catal., B 2021, 293, 120178 10.1016/j.apcatb.2021.120178.

[ref20] He L.; Hu S.; Jiang L.; Liao G.; Zhang L.; Han H.; Chen X.; Wang Y.; Xu K.; Su S.; Xiang J. Co-Production of Hydrogen and Carbon Nanotubes from the Decomposition/Reforming of Biomass-Derived Organics over Ni/α-Al_2_O_3_ Catalyst: Performance of Different Compounds. Fuel 2017, 210 (May), 307–314. 10.1016/j.fuel.2017.08.080.

[ref21] Hu X.; Lu G. Investigation of the Steam Reforming of a Series of Model Compounds Derived from Bio-Oil for Hydrogen Production. Appl. Catal., B 2009, 88 (3–4), 376–385. 10.1016/j.apcatb.2008.10.021.

[ref22] Venkataraman A.; Amadi E. V.; Chen Y.; Papadopoulos C. Carbon Nanotube Assembly and Integration for Applications. Nanoscale Res. Lett. 2019, 14 (1), 220 10.1186/s11671-019-3046-3. 31263975 PMC6603253

[ref23] Oriňáková R.; Oriňák A. Recent Applications of Carbon Nanotubes in Hydrogen Production and Storage. Fuel 2011, 90 (11), 3123–3140. 10.1016/j.fuel.2011.06.051.

[ref24] Phung T. K.; Pham T. L. M.; Nguyen A.-N. T.; Vu K. B.; Giang H. N.; Nguyen T.; Huynh T. C.; Pham H. D. Effect of Supports and Promoters on the Performance of Ni-Based Catalysts in Ethanol Steam Reforming. Chem. Eng. Technol. 2020, 43 (4), 672–688. 10.1002/ceat.201900445.

[ref25] Boudadi K.; Bellifa A.; Márquez-Álvarez C.; Cortés Corberán V. Nickel Catalysts Promoted with Lanthanum for Ethanol Steam Reforming: Influence of Support and Treatment on Activity. Appl. Catal., A 2021, 619, 118141 10.1016/j.apcata.2021.118141.

[ref26] Soykal I. I.; Sohn H.; Ozkan U. S. Effect of Support Particle Size in Steam Reforming of Ethanol over Co/CeO_2_ Catalysts. ACS Catal. 2012, 2 (11), 2335–2348. 10.1021/cs3004159.

[ref27] Xu W.; Liu Z.; Johnston-Peck A. C.; Senanayake S. D.; Zhou G.; Stacchiola D.; Stach E. A.; Rodriguez J. A. Steam Reforming of Ethanol on Ni/CeO_2_: Reaction Pathway and Interaction between Ni and the CeO_2_ Support. ACS Catal. 2013, 3 (5), 975–984. 10.1021/cs4000969.

[ref28] Pu J.; Luo Y.; Wang N.; Bao H.; Wang X.; Qian E. W. Ceria-Promoted Ni@Al_2_O_3_ Core-Shell Catalyst for Steam Reforming of Acetic Acid with Enhanced Activity and Coke Resistance. Int. J. Hydrogen Energy 2018, 43 (6), 3142–3153. 10.1016/j.ijhydene.2017.12.136.

[ref29] Xiao Z.; Wu C.; Wang L.; Xu J.; Zheng Q.; Pan L.; Zou J.; Zhang X.; Li G. Boosting Hydrogen Production from Steam Reforming of Ethanol on Nickel by Lanthanum Doped Ceria. Appl. Catal., B 2021, 286, 119884 10.1016/j.apcatb.2021.119884.

[ref30] Zhurka M. D.; Lemonidou A. A.; Kechagiopoulos P. N. Elucidation of Metal and Support Effects during Ethanol Steam Reforming over Ni and Rh Based Catalysts Supported on (CeO_2_)-ZrO_2_-La_2_O_3_. Catal. Today 2021, 368, 161–172. 10.1016/j.cattod.2020.03.020.

[ref31] Shtyka O.; Dimitrova Z.; Ciesielski R.; Kedziora A.; Mitukiewicz G.; Leyko J.; Maniukewicz W.; Czylkowska A.; Maniecki T. Steam Reforming of Ethanol for Hydrogen Production: Influence of Catalyst Composition (Ni/Al_2_O_3_, Ni/Al_2_O_3_–CeO_2_, Ni/Al_2_O_3_–ZnO) and Process Conditions. React. Kinet., Mech. Catal. 2021, 132 (2), 907–919. 10.1007/s11144-021-01945-6.

[ref32] Montero C.; Remiro A.; Valle B.; Oar-Arteta L.; Bilbao J.; Gayubo A. G. Origin and Nature of Coke in Ethanol Steam Reforming and Its Role in Deactivation of Ni/La_2_O_3_-αAl_2_O_3_ Catalyst. Ind. Eng. Chem. Res. 2019, 58 (32), 14736–14751. 10.1021/acs.iecr.9b02880.

[ref33] Montero C.; Remiro A.; Benito P. L.; Bilbao J.; Gayubo A. G. Optimum Operating Conditions in Ethanol Steam Reforming over a Ni/La_2_O_3_-αAl_2_O_3_ Catalyst in a Fluidized Bed Reactor. Fuel Process. Technol. 2018, 169, 207–216. 10.1016/j.fuproc.2017.10.003.

[ref34] Montero C.; Valle B.; Bilbao J.; Gayubo A. G. Analysis of Ni/La_2_O_3_-αAl_2_O_3_ Catalyst Deactivation by Coke Deposition in the Ethanol Steam Reforming. Chem. Eng. Trans. 2014, 37 (2), 481–486.

[ref35] Elias K. F. M.; Lucrédio A. F.; Assaf E. M. Effect of CaO Addition on Acid Properties of Ni-Ca/Al_2_O_3_ Catalysts Applied to Ethanol Steam Reforming. Int. J. Hydrogen Energy 2013, 38 (11), 4407–4417. 10.1016/j.ijhydene.2013.01.162.

[ref36] Dou B.; Zhang H.; Cui G.; Wang Z.; Jiang B.; Wang K.; Chen H.; Xu Y. Hydrogen Production by Sorption-Enhanced Chemical Looping Steam Reforming of Ethanol in an Alternating Fixed-Bed Reactor: Sorbent to Catalyst Ratio Dependencies. Energy Convers. Manage. 2018, 155, 243–252. 10.1016/j.enconman.2017.10.075.

[ref37] Menendez R. B.; Graschinsky C.; Amadeo N. E. Sorption-Enhanced Ethanol Steam Reforming Process in a Fixed-Bed Reactor. Ind. Eng. Chem. Res. 2018, 57 (34), 11547–11553. 10.1021/acs.iecr.8b01657.

[ref38] Barroso M. N.; Gomez M. F.; Arrúa L. A.; Abello M. C. Reactivity of Aluminum Spinels in the Ethanol Steam Reforming Reaction. Catal. Lett. 2006, 109 (1–2), 13–19. 10.1007/s10562-006-0051-9.

[ref39] Remiro A.; Arandia A.; Oar-Arteta L.; Bilbao J.; Gayubo A. G. Regeneration of NiAl_2_O_4_ Spinel Type Catalysts Used in the Reforming of Raw Bio-Oil. Appl. Catal., B 2018, 237, 353–365. 10.1016/j.apcatb.2018.06.005.

[ref40] Arandia A.; Remiro A.; García V.; Castaño P.; Bilbao J.; Gayubo A. Oxidative Steam Reforming of Raw Bio-Oil over Supported and Bulk Ni Catalysts for Hydrogen Production. Catalysts 2018, 8 (8), 322 10.3390/catal8080322.

[ref41] Nuñez Meireles M.; Alonso J. A.; Fernández Díaz M. T.; Cadús L. E.; Aguero F. N. Ni Particles Generated in Situ from Spinel Structures Used in Ethanol Steam Reforming Reaction. Mater. Today Chem. 2020, 15, 100213 10.1016/j.mtchem.2019.100213.

[ref42] García-Gómez N.; Valle B.; Valecillos J.; Remiro A.; Bilbao J.; Gayubo A. G. Feasibility of Online Pre-Reforming Step with Dolomite for Improving Ni Spinel Catalyst Stability in the Steam Reforming of Raw Bio-Oil. Fuel Process. Technol. 2021, 215, 106769 10.1016/j.fuproc.2021.106769.

[ref43] Valle B.; García-Gómez N.; Arandia A.; Remiro A.; Bilbao J.; Gayubo A. G. Effect of Phenols Extraction on the Behavior of Ni-Spinel Derived Catalyst for Raw Bio-Oil Steam Reforming. Int. J. Hydrogen Energy 2019, 44 (25), 12593–12603. 10.1016/j.ijhydene.2018.12.057.

[ref44] Akande A. J.; Idem R. O.; Dalai A. K. Synthesis, Characterization and Performance Evaluation of Ni/Al_2_O_3_ Catalysts for Reforming of Crude Ethanol for Hydrogen Production. Appl. Catal., A 2005, 287 (2), 159–175. 10.1016/j.apcata.2005.03.046.

[ref45] Jiménez-González C.; Boukha Z.; De Rivas B.; Delgado J. J.; Cauqui M. Á.; González-Velasco J. R.; Gutiérrez-Ortiz J. I.; López-Fonseca R. Structural Characterisation of Ni/Alumina Reforming Catalysts Activated at High Temperatures. Appl. Catal., A 2013, 466, 9–20. 10.1016/j.apcata.2013.06.017.

[ref46] Arandia A.; Remiro A.; Valle B.; Bilbao J.; Gayubo A. G. Deactivation of Ni Spinel Derived Catalyst during the Oxidative Steam Reforming of Raw Bio-Oil. Fuel 2020, 276, 117995 10.1016/j.fuel.2020.117995.

[ref47] Hasan M.; Drazin J.; Dey S.; Castro R. H. R. Synthesis of Stoichiometric Nickel Aluminate Spinel Nanoparticles. Am. Mineral. 2015, 100 (2–3), 652–657. 10.2138/am-2015-4997.

[ref48] Santamaria L.; Lopez G.; Arregi A.; Amutio M.; Artetxe M.; Bilbao J.; Olazar M. Stability of Different Ni Supported Catalysts in the In-Line Steam Reforming of Biomass Fast Pyrolysis Volatiles. Appl. Catal., B 2019, 242, 109–120. 10.1016/j.apcatb.2018.09.081.

[ref49] Gayubo A. G.; Aguayo A. T.; Atutxa A.; Prieto R.; Bilbao J. Role of Reaction-Medium Water on the Acidity Deterioration of a HZSM-5 Zeolite. Ind. Eng. Chem. Res. 2004, 43 (17), 5042–5048. 10.1021/ie0306630.

[ref50] Morales-Marín A.; Ayastuy J. L.; Iriarte-Velasco U.; Gutiérrez-Ortiz M. A. Nickel Aluminate Spinel-Derived Catalysts for the Aqueous Phase Reforming of Glycerol: Effect of Reduction Temperature. Appl. Catal., B 2019, 244, 931–945. 10.1016/j.apcatb.2018.12.020.

[ref51] Song K. H.; Jeong S. K.; Jeong B. H.; Lee K.-Y.; Kim H. J. Effect of the Ni/Al Ratio on the Performance of NiAl_2_O_4_ Spinel-Based Catalysts for Supercritical Methylcyclohexane Catalytic Cracking. Catalysts 2021, 11 (3), 323 10.3390/catal11030323.

[ref52] Choya A.; de Rivas B.; Gutiérrez-Ortiz J. I.; González-Velasco J. R.; López-Fonseca R. Synthesis, Characterization and Kinetic Behavior of Supported Cobalt Catalysts for Oxidative after-Treatment of Methane Lean Mixtures. Materials 2019, 12 (19), 3174 10.3390/ma12193174. PMC6804103 31569775

[ref53] Boukha Z.; Jiménez-González C.; de Rivas B.; González-Velasco J. R.; Gutiérrez-Ortiz J. I.; López-Fonseca R. Synthesis, Characterisation and Performance Evaluation of Spinel-Derived Ni/Al_2_O_3_ Catalysts for Various Methane Reforming Reactions. Appl. Catal., B 2014, 158–159, 190–201. 10.1016/j.apcatb.2014.04.014.

[ref54] Di Michele A.; Dell’Angelo A.; Tripodi A.; Bahadori E.; Sànchez F.; Motta D.; Dimitratos N.; Rossetti I.; Ramis G. Steam Reforming of Ethanol over Ni/MgAl_2_O_4_ Catalysts. Int. J. Hydrogen Energy 2019, 44 (2), 952–964. 10.1016/j.ijhydene.2018.11.048.

[ref55] Bonnin A.; Comparot J. D.; Pouilloux Y.; Coupard V.; Uzio D.; Pinard L. Mechanisms of Aromatization of Dilute Ethylene on HZSM-5 and on Zn/HZSM-5 Catalysts. Appl. Catal., A 2021, 611, 117974 10.1016/j.apcata.2020.117974.

[ref56] Zhu Q.; Kondo J. N.; Inagaki S.; Tatsumi T. Catalytic Activities of Alcohol Transformations over 8-Ring Zeolites. Top. Catal. 2009, 52 (9), 1272–1280. 10.1007/s11244-009-9272-7.

[ref57] Nash C. P.; Ramanathan A.; Ruddy D. A.; Behl M.; Gjersing E.; Griffin M.; Zhu H.; Subramaniam B.; Schaidle J. A.; Hensley J. E. Mixed Alcohol Dehydration over Brønsted and Lewis Acidic Catalysts. Appl. Catal., A 2016, 510, 110–124. 10.1016/j.apcata.2015.11.019.

[ref58] Gayubo A. G.; Valle B.; Aramburu B.; Montero C.; Bilbao J. Kinetic Model Considering Catalyst Deactivation for the Steam Reforming of Bio-Oil over Ni/La_2_O_3_-αAl_2_O_3_. Chem. Eng. J. 2018, 332, 192–204. 10.1016/j.cej.2017.09.063.

[ref59] Setiabudi H. D.; Aziz M. A. A.; Abdullah S.; Teh L. P.; Jusoh R. Hydrogen Production from Catalytic Steam Reforming of Biomass Pyrolysis Oil or Bio-Oil Derivatives: A Review. Int. J. Hydrogen Energy 2020, 45 (36), 18376–18397. 10.1016/j.ijhydene.2019.10.141.

