# Supplementary material for: Insights into the Reaction Routes for H2 Formation in the Ethanol Steam Reforming on a Catalyst Derived from NiAl2O4 Spinel
Source: Energy Fuels. 2021 Jul 29;35(21):17197–211. doi: 10.1021/acs.energyfuels.1c01670 (PMC8573826; doi:10.1021/acs.energyfuels.1c01670)
Supplement: Supplementary file 1 — ef1c01670_si_001.pdf [file ef1c01670_si_001.pdf]

Insights into the reaction routes for H_2 formation in the ethanol steam reforming on a catalyst derived from $NiAl_2O_4$ spinel

José Valecillos*, Sergio Iglesias-Vázquez, Leire Landa, Aingeru Remiro, Javier Bilbao, Ana G. Gayubo*

Department of Chemical Engineering, University of the Basque Country (UPV/EHU) P.O. Box 644, Bilbao, 48080 Spain

(*) Corresponding author(s): anaguadalupe.gayubo@ehu.eus, jose.valecillos@ehu.eus

This document contains supplementary results.

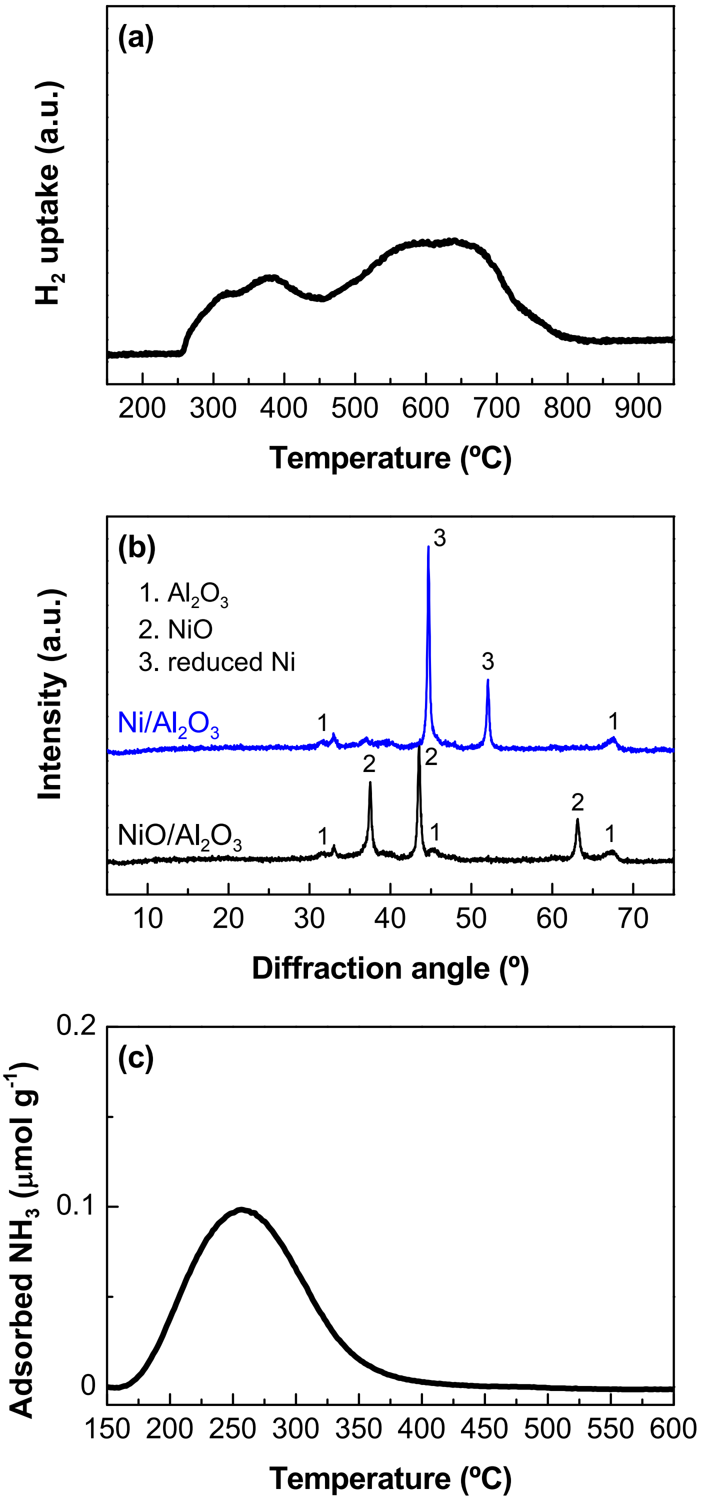

Figure S1. Characterization results of the catalyst prepared by impregnation: (a) TPR profile of NiO/Al_2O_3 precursor, (b) XRD patterns of NiO/Al_2O_3 precursor and corresponding derived Ni/Al_2O_3 catalyst, and (c) NH_3-TPD profile of the Ni/Al_2O_3 catalyst.

Table S1. Textural properties of the NiO/Al_2O_3 precursor and Ni/Al_2O_3 catalyst prepared by wet impregnation.

**Property**	**NiO/Al_2O_3**	**Ni/Al_2O_3**
S_{BET} (m^2 g^{-1})	61.8	58.4
V_{pore} (cm^3 g^{-1})	0.237	0.251
Pore size (nm)	15.3	17.1

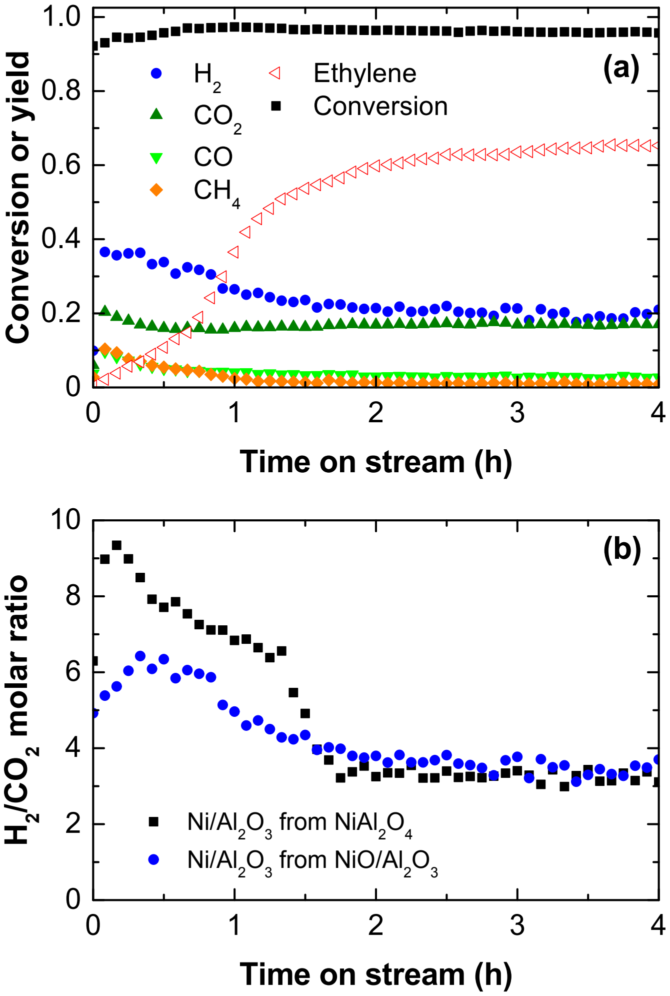

Figure S2. Evolution with time on stream of the ethanol conversion and product yield (a) and H_2/CO_2 molar ratio (b) in the ESR on a Ni/Al_2O_3 catalyst prepared by impregnation, at a space-time of 0.025 h, steam/ethanol/N_2 ratio of 3/1/16 and 500 ºC (values of H_2/CO_2 molar ratio for spinel derived catalysts are included in graph (b) for comparison).

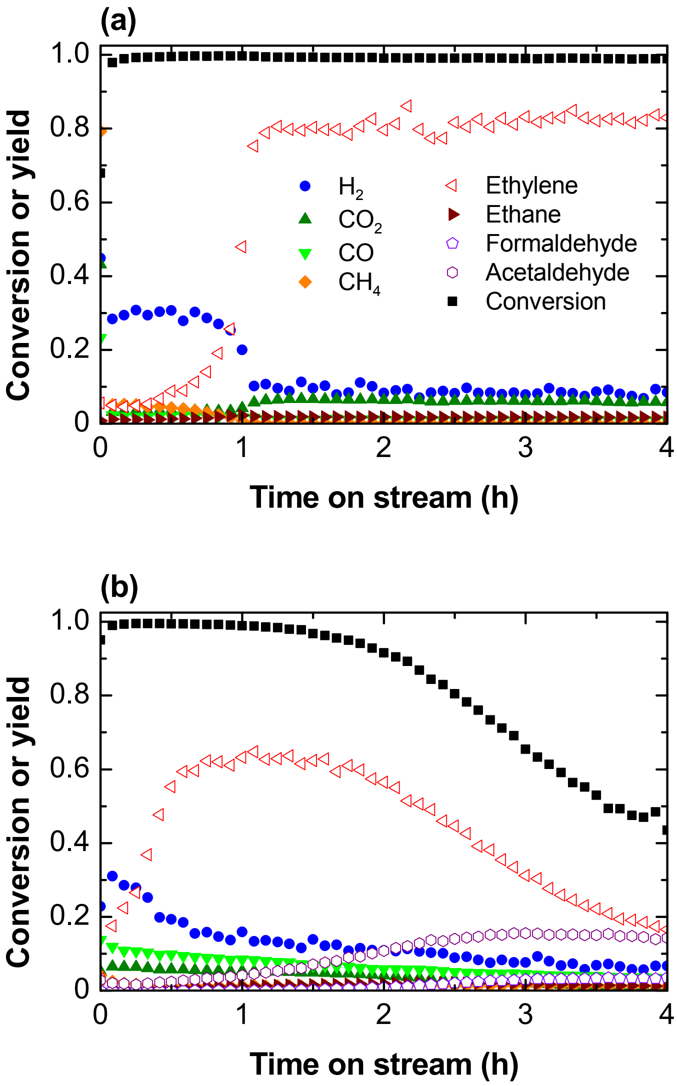

Figure S3. Evolution with time on stream of the ethanol conversion and product yields for the ethanol decomposition at (a) 500 ºC and (b) 600 ºC. Reaction conditions: 500 or 600 ºC, space-time = 0.025 h, steam/ethanol/N_2 molar ratio of 0/1/19.

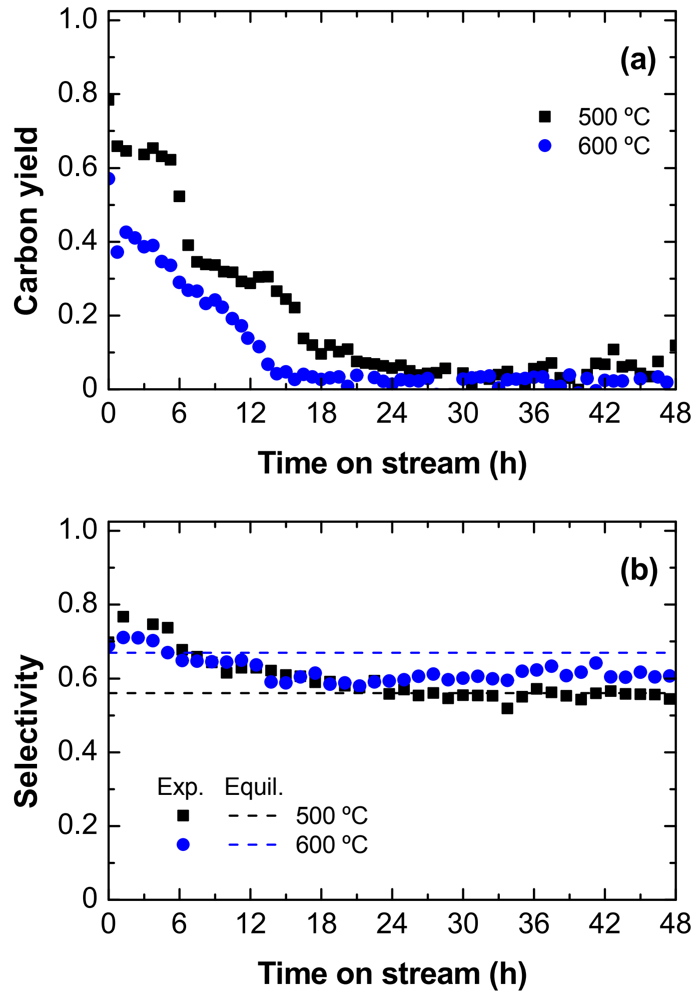

Figure S4. Evolution with time on stream of solid carbon yield estimated from carbon balance (a) and the H_2 selectivity (b) for the ESR at 500 ºC and 600 ºC. Reaction conditions: space-time = 0.1 h, steam/ethanol/N_2 molar ratio of 3/1/16.

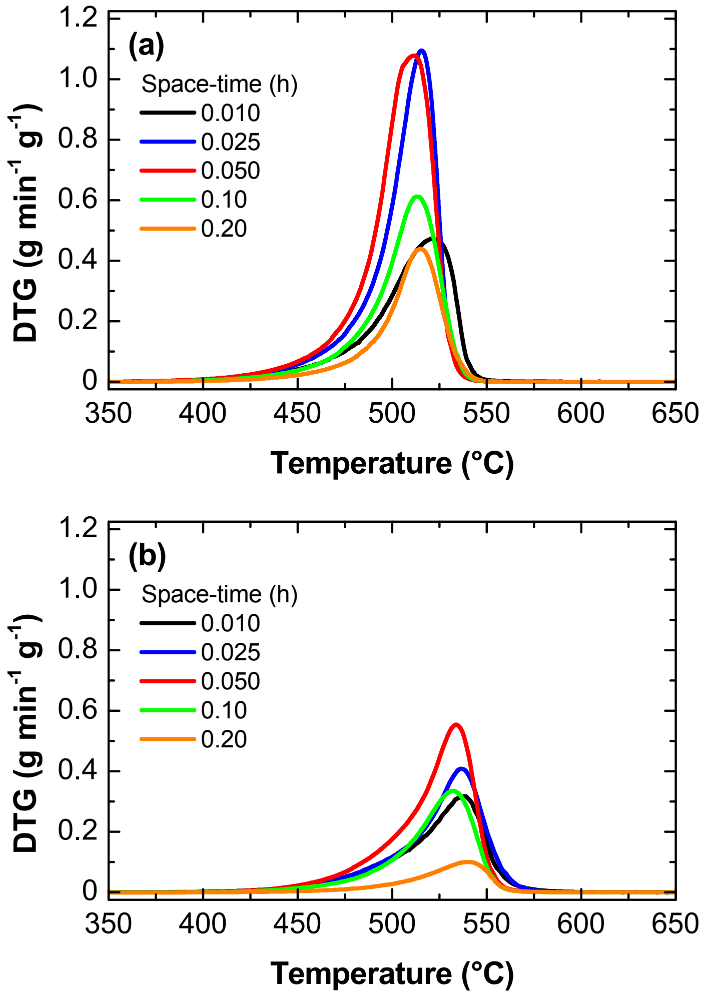

Figure S5. TPO profiles of carbon formed in the ESR after 4 h at different space-time values at (a) 500 and (b) 600 ºC (corresponding to the results described in Figure 2).

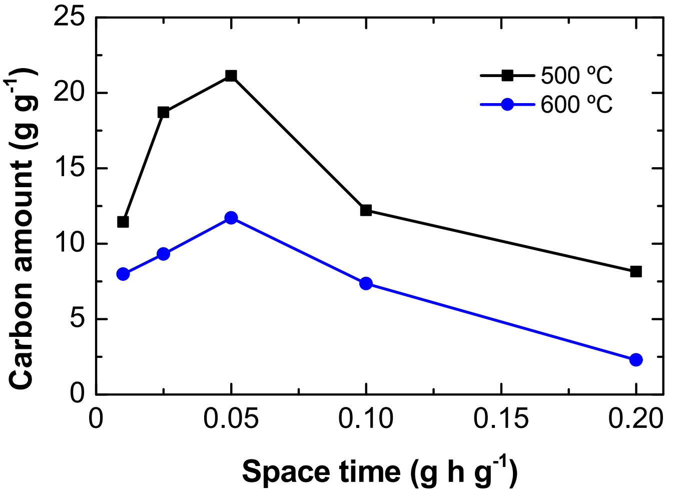

Figure S6. Evolution with space-time of the carbon amount formed in 4 h for the ESR at 500 ºC and 600 ºC.
